# A novel lineage of osteoprogenitor cells with dual epithelial and mesenchymal properties govern maxillofacial bone homeostasis and regeneration after MSFL

**DOI:** 10.1038/s41422-022-00687-x

**Published:** 2022-07-12

**Authors:** Yuteng Weng, Haicheng Wang, Di Wu, Shuyu Xu, Xiaofan Chen, Jie Huang, Yanhuizhi Feng, Lin Li, Zuolin Wang

**Affiliations:** Department of Oral Implantology & Department of Oral and Maxillofacial Surgery, Stomatological Hospital and Dental School of Tongji University, Shanghai Engineering Research Center of Tooth Restoration and Regeneration, Shanghai, China

**Keywords:** Transdifferentiation, Bioinformatics, Regeneration

## Abstract

Bone regeneration originates from proliferation and differentiation of osteoprogenitors via either endochondral or intramembranous ossification; and the regeneration capacities decline with age and estrogen loss. Maxillary sinus floor lifting (MSFL) is a commonly used surgical procedure for guiding bone regeneration in maxilla. Radiographic analysis of 1210 clinical cases of maxilla bone regeneration after MSFL revealed that the intrasinus osteogenic efficacy was independent of age and gender, however; and this might be related to the Schneiderian membrane that lines the sinus cavity. In view of the particularity of this biological process, our present study aimed to elucidate the underlying mechanism of MSFL-induced bone regeneration. We first established a murine model to simulate the clinical MSFL. By single-cell RNA-sequencing and flow cytometry-based bulk RNA-sequencing, we identified a novel *Krt14*^*+*^*Ctsk*^*+*^ subset of cells that display both epithelial and mesenchymal properties and the transcriptomic feature of osteoprogenitors. Dual recombinases-mediated lineage tracing and loss-of-function analyses showed that these *Krt14*^*+*^*Ctsk*^*+*^ progenitors contribute to both MSFL-induced osteogenesis and physiological bone homeostasis by differentiating into *Krt14*^–^*Ctsk*^*+*^ descendants which show robust osteogenic capacity. In addition, we detected a similar population of *Krt14*^*+*^*Ctsk*^*+*^ cells in human samples of Schneiderian membrane, which show a highly similar osteogenic potential and transcriptomic feature to the corresponding cells in mice. The identification of this *Krt14*^*+*^*Ctsk*^*+*^ population, featured by osteoprogenitor characteristics and dual epithelial–mesenchymal properties, provides new insight into the understanding of bone regeneration and may open more possibilities for clinical applications.

## Introduction

Injury-induced bone regeneration is a basic physical response in order to maintain skeletal function. It is a sophisticated pathophysiological process that relies on the complex regulation of multiple cellular networks.^1–3^ As the driver of bone regeneration, osteoprogenitors differentiate into osteoblasts, and by either intramembranous or endochondral ossification, orchestrate the regeneration process and maintain the homeostatic bone turnover in a timely and robust manner.^4–6^

Previous studies have depicted a relatively complete osteogenic network in axial and appendicular bones, which involves diverse subsets of skeletal progenitors, including bone marrow stromal cells,^7–13^ chondrocytes^14,15^ and periosteal stromal cells.^16–18^ Osteoprogenitors with mesenchymal origin are known to locate at the periosteum, bone marrow and growth plate. The regenerative capacity of skeletal progenitors in long bones is significantly affected by age and estrogen level. It declines during aging and post menopause, which has been verified by multiple researches.^19–21^

Craniomaxillofacial bones are a part of the skeletal system. Due to the special anatomical structures and physiological features of craniomaxillofacial bones, the function of osteoprogenitor cells in craniomaxillofacial bone tissue is different from that in the long bone.^22,23^ Recent evidence shows that the major osteoprogenitor cells in the cranial region are primarily located within the periosteum or suture mesenchyme of the skull.^22,24^ So far, four markers have been verified to label osteoprogenitor cells in the suture, including Cathepsin K (*Ctsk*),^16^ *Prrx1*,^24^ *Gli1*,^22^ and *Axin2*.^25^

The maxillofacial bones including maxilla and nasal bone are important skeletal structures for the contour and function of the middle face. Maxillofacial bone defects caused by different reasons can seriously affect facial profile, as well as physiological functions including speech, mastication and swallowing.^26,27^ There is a special anatomical structure in the body of maxilla, known as maxillary sinus, whose cavity is lined by the Schneiderian membrane (SM), an extension of respiratory epithelium.^28–30^ It has been well documented that a type of guided bone regeneration technique used clinically, called maxillary sinus floor lifting (MSFL),^31^ which stably elevates the SM through insertion of dental implants, is capable of inducing intrasinus new bone formation with the regenerative bone volume closely related to the membrane elevating height. Based on the radiographic analysis of 1210 clinical MSFL cases from our department, it was observed that the osteogenic efficacy of MSFL was independent of age or gender, which is different from the phenomena described in long bone repair. As far as we know, the underlying mechanism involved in maxillary sinus osteogenesis has not yet been explicated.

Two methods had been reported to establish the MSFL-like animal models, the transnasal^32,33^ and transalveolar approaches.^34^ In this study, we established two murine models to simulate the clinical MSFL through both approaches mentioned above. Using histologic and Micro-CT-based radiographic analyses, it was observed that peri-implant bone regeneration was achieved in both nasal cavity and maxillary sinus. By bulk RNA sequencing (RNA-seq), we found high similarity of transcriptomic features between the respiratory epithelium-derived nasal mucosa (NM) and SM. However, the limited space of mouse maxillary sinus does not allow retrieving adequate sample for the following high-throughput sequencing. Therefore, the MSFL-like model generated via nasal bone was finally used to collect regenerated bone tissue for subsequent analysis. By using single-cell RNA-sequencing (scRNA-seq) to dissect the cellular composition and lineage hierarchy within heterogenous cell population, we identified a unique cell cluster (*Krt14*^*+*^*Ctsk*^*+*^), co-expressing epithelial (*Krt14*) and mesenchymal (*Ctsk*) markers. By fluorescence-activated cell sorting (FACS)-based bulk RNA-seq, this cluster was proven to share transcriptomic features with osteoprogenitors and dual epithelial–mesenchymal properties. By dual recombinase-mediated lineage tracing and loss-of-function analyses, we showed that the *Krt14*^*+*^*Ctsk*^*+*^ descendants contributed to both MSFL-induced osteogenesis and physiological bone homeostasis. In addition to nasal cavity, *Krt14*^*+*^*Ctsk*^*+*^ progenitors were also found in the regenerated tissue in the mouse MSFL model generated via maxilla. The current work reveals that the *Krt14*^*+*^*Ctsk*^*+*^ cells serve as a novel lineage of osteoprogenitor cells in maxillofacial bone regeneration.

## Results

### Clinical MSFL cohorts and establishment of MSFL mouse model

Between January 2010 and December 2020, 1210 patients (565 men and 645 women) aged between 18 and 75 years, were enrolled in this study. All patients were treated with MSFL at the Department of Oral Implantology, Stomatological Hospital of Tongji University. During MSFL, the hSM was gently lifted by slow insertion of a dental implant, and a secluded space was created between the sinus floor and the hSM with blood clot filling. New osseous tissue was regenerated in this gap and the vertical bone volume substantially increased (Supplementary information, Fig. S1a). Cone-beam computed tomography (CBCT) images demonstrated the restoration of edentulous posterior maxilla (Supplementary information, Fig. S1b). Statistical analysis indicated that the osteogenic efficacy reached up to 0.86 ± 0.22, and no statistically significant difference of osteogenic efficacy was found among patients of different ages (*P* = 0.64) or gender (*P* = 0.80) (Supplementary information, Fig. S1c, d). However, aging^19,20^ and menopause^21^ have been proven to impair the osteogenic capacity of osteoprogenitors in long bones.

**Fig. 1:**
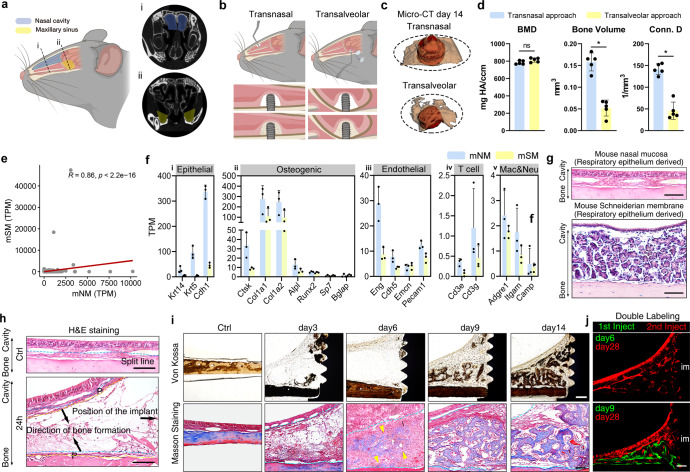
The establishment of a mouse MSFL model. **a** Schematic diagram of the anatomical positions of murine nasal cavity and maxillary sinus, indicated by light blue and yellow, respectively. The corresponding coronal X-ray sections were displayed. (i) The section of nasal cavity. (ii) The section of maxillary sinus. **b** Schematic diagrams of murine MSFL models generated via transnasal and transalveolar approaches. **c** The representative Micro-CT reconstruction images of MSFL-induced osteogenesis on day 14 post surgery. *n* = 5 from 3 independent experiments. **d** Statistical analysis of bone mineral density (BMD), absolute bone volume and connective densities of the regenerative bones of MSFL by two different approaches. *n* = 5 from 3 independent experiments. *P < 0.05; Student’s *t*-test was used. **e** Correlation analysis of mSM and mNM at transcriptomic level. TPM reads of genes were analyzed by Spearman correlation analysis. *R* = 0.86, *P* < 2.2e–16. **f** Comparison of TPM of T cell-, macrophage-, neutrophil-, epithelial cell-, osteolineage cell-, and endothelial cell-specific markers in mSM and mNM. Error bars indicate SEM. **g** Representative H&E staining images showing the histological structures of mNM and mSM. Scale bar, 100 μm. **h** Representative H&E staining images showing the histological variation observed before and 24 h after MSFL. The split line is shown as a blue dotted line. The right black arrow depicts the implant location. The two left ones indicate the direction of bone formation. The region between the yellow and blue dashed lines indicates the layers of proliferating progenitor cells (P). Scale bar, 100 μm. **i** Representative Von Kossa and Masson staining images showing the changes of the calcification degrees and microstructures of the newly formed tissue before and 3, 6, 9, 14 days after MSFL. In Von Kossa staining images, brown-stained regions indicate calcified tissue. In Masson staining images, blue-stained regions represent immature collagen (osteoid or newly-formed woven bone); red-stained regions represent mature collagen (fibrous connective tissue or mature lamellar bone). Yellow arrowheads indicate that osteoid was formed on day 6. Scale bar, 50 μm. **j** Representative double labeling images showing dynamic osseous deposition. The 1st injection of calcein on day 6 showed no sign of mineralization (top). Positive calcein fluorescent bands (green) could be found when the 1st injection changed to day 9 (bottom), suggesting that calcium deposition started on day 9. im, implant; scale bar, 50 μm. For **h**–**j**, *n* = 3 per condition from three independent experiments.

To explore the potential mechanism of MSFL-induced osteogenesis, we started this study by establishing an animal model recapitulating the MSFL in human. We compared the animal models of MSFL generated by two commonly used approaches, the transnasal and transalveolar ones, separately (Fig. 1a, b). From the aspect of anatomy, the mouse nasal cavity was much larger than the maxillary sinus which guaranteed the insertion of longer mini-implants (0.8 mm), accompanied by relatively abundant regenerative tissues available (Fig. 1a). By Micro-CT-based radiographic analyses, both MSFL approaches could induce bone regeneration on day 14 post surgery, while the transnasal one would yield significantly higher amount of bone volume, which was in line with the anatomical basis (Fig. 1c, d). The bulk RNA-seq and morphology analysis were then carried out to evaluate the similarities in both transcriptomic and histological features of mouse NM (mNM) and SM (mSM). The results indicated a significant correlation between the transcriptomes of mNM and mSM (*R* = 0.86, *P* < 2.2e–16) (Fig. 1e). To clarify the cell compositions of mNM and mSM, transcripts per million (TPM) reads of different cell-specific markers were compared accordingly (Fig. 1f). Collectively, higher expressions of epithelial markers were observed (*Krt14*, *Krt5* and *Cdh1)* in mNM, while expressions of markers concerning osteogenic cell components (*Col1a1*, *Col1a2*, *Alpl*, *Runx2*, and *Sp7*) and immune cells (T cell, macrophage and neutrophil) were similar between the two structures (Fig. 1f). Histologically, given the identical origins of respiratory epithelia,^34^ both mNM and mSM were constituted with the epithelium and connective tissue layer full of glands, with the latter containing significantly richer maxillary glands (Fig. 1g).

Although it was observed that mNM displayed superior efficacy in inducing regenerative tissues and had similar features to mSM, the comparisons of transcriptomic features between mNM and human SM (hSM) was also performed to ensure the usability of this approach before carrying out formal studies. By comparing the TPM of homologous protein-coding genes between human and mouse, we found a significant correlation between transcriptomes of hSM and mNM (*R* = 0.65, *P* < 2.2e–16; Supplementary information, Fig. S1e). To clarify the cell compositions of hSM and mNM, similar comparisons were carried out. Collectively, higher expression levels of endothelial cell (*Eng* and *Pecam1*), osteolineage cell (*Col1a1*, *Col1a2*, *Alpl*, *Runx2*, and *Sp7*), and T cell (*Cd3e*) specific markers could be found in hSM (Supplementary information, Fig. S1f).

Based on the similar anatomical and transcriptomic features shared by hSM and mNM, we chose the murine MSFL model generated by the transnasal approach in the following studies. To verify whether MSFL in mice could induce de novo bone formation recapitulating that in human, histological assays were performed. Hematoxylin and Eosin (H&E) staining carried out 24 h after operation showed that the mNM was split into two layers from the middle (Fig. 1h). Blood cells and fibrin networks were distributed between the two split layers on day 3. Proliferation of fibroblast-like cells and osteoid deposition were apparent on day 6, but no mineralized tissue was observed. On days 9 and 14, woven bone filled this area, and mineral density increased significantly over time (Fig. 1i). To assess the osteogenesis dynamics, double labeling assay indicated that calcium deposition started on day 9 with calcein signal shown in the elevated region (Fig. 1j). Taken together, these data show that MSFL in mouse models could also induce local bone formation.

**Fig. 2:**
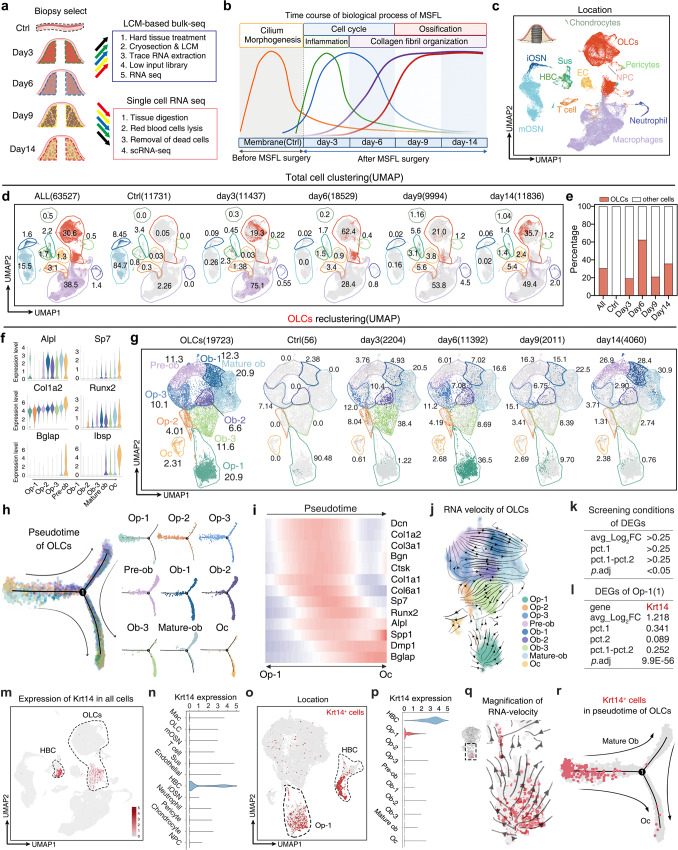
Identification of *Krt14*^*+*^ OLCs by scRNA-seq in a mouse MSFL model. **a** Workflow of high-throughput sequencing including LCM-based bulk RNA-seq and scRNA-seq. **b** Time course analysis of MSFL-induced sequential biological process before and 3, 6, 9, and 14 days after surgery. **c** Distribution of 63,527 cells from mNM (Ctrl) and newly-formed tissue from postoperative days 3, 6, 9, and 14. In total, 12 clusters were visualized by UMAP. EC, endothelial cell; iOSN, immature olfactory sensory neuron; mOSN, mature olfactory sensory neuron; NPC, neuron progenitor cell; Sus, sustentacular cell. **d** Distribution and proportion of 12 clusters visualized by UMAP projection; color coded by each cluster, with the proportion labeled aside; cells at all time points (left), sequential layout of cells at each time point (right). **e** Histogram of OLC proportion variation originated from **d**. OLCs at all time points: 30.6%; OLCs in preoperative mNM (Ctrl): 0.06%; on day 3: 19.3%; on day 6: 62.4%; on day 9: 21.0%; on day 14: 35.7%. **f** Violin plots showing the log-normalized expression levels of curated feature genes in 9 re-clustered subsets of OLCs. Op osteoprogenitor, Ob osteoblast, Oc osteocyte. **g** Distribution of 19,723 re-clustered OLCs. In total, 9 subclusters were visualized by UMAP projection. OLCs at all time points (left), sequential layout of OLCs at each time point (right), color coded by subclusters, with the proportion labeled aside. **h** Pseudotime lineage trajectory analysis demonstrating the relationships of subclusters among OLCs, color coded by subclusters. **i** Heatmap showing variation of osteogenic-related gene expression in pseudotime. **j** RNA velocity of OLCs estimated from unspliced and spliced transcripts of nearby cells and visualized on the UMAP projection; color coded by clusters. **k** Screening conditions for DEGs of the Op-1 compared to the rest of the OLCs. **l** DEG list of Op-1. *Krt14*, a classic epithelial marker, was expressed by Op-1 (Log_2_FC = 1.218, *P* = 9.9e–56). **m** Expression of *Krt14* visualized on UMAP projection of all cells, color coded by expression level of *Krt14*. **n** Violin plots generated from **m**, showing log-normalized expression values of *Krt14*. **o** Expression of *Krt14* visualized on UMAP projection of OLCs and HBCs, color coded by expression level of *Krt14*. **p** Violin plots generated from **n**, showing log-normalized expression values of *Krt14*. **q** RNA velocity of Op-1 visualized on the magnified UMAP projection; color coded by expression level of *Krt14*. **r** Distribution of *Krt14*^*+*^ cells in the pseudotime trajectory of OLCs; color coded by the expression level of *Krt14*.

To describe MSFL-induced sequential changes at transcriptomic level, preoperative mNM (Ctrl) and the regenerative tissues on postoperative days 3, 6, 9, and 14 were subjected to laser captured microdissection-based bulk RNA-seq (LCM-based bulk RNA-seq; Fig. 2a). Based on time course analysis (Supplementary information, Fig. S2a) and weighted gene co-expression network analysis (WGCNA) (Supplementary information, Fig. S2b), characteristic genes were divided into six major modules, with each one showing a unique expression time course. Then, these modules were subjected to gene ontology-biology process (GO-BP) (Supplementary information, Fig. S2c) and Kyoto Encyclopedia of Genes and Genomes (KEGG) enrichment analyses (Supplementary information, Fig. S2d). Preoperative mNM (Ctrl) samples were characterized with cilium morphogenesis, which was in accordance with its respiratory epithelium identity. Samples from days 3 and 6 were featured by proliferation and osteogenesis, and enriched in major signaling pathways related to proliferation. Samples from days 9 and 14 were enriched in osteogenesis and related signaling pathways (Fig. 2b). These results suggested that MSFL initiated a cascade of well-organized biological processes starting from proliferation to the terminal osteogenesis.

Taken together, as indicated by the superior regenerative capability and similar anatomical and transcriptomic features of hSM and mNM, we established a murine MSFL model by the transnasal approach to elucidate the biological nature underlying the bone regeneration. Notable de novo osteogenesis was induced and verified at histological and transcriptome levels.

### Identification of osteoprogenitor cells with epithelial characteristics by scRNA-seq in MSFL-induced osteogenesis

To dissect the MSFL-induced biological reactions at single-cell resolution, preoperative mNM (Ctrl) and the regenerative tissues on postoperative days 3, 6, 9, and 14 were subjected to scRNA-seq to clarify the cellular composition and lineage hierarchy (Fig. 2a). After enzymatic digestion, quality control and doublet exclusion, we obtained 63,527 single cells. After uniform manifold approximation and projection (UMAP) clustering, cells were divided into 12 clusters by different cell-specific markers according to the previous study^35^ (Fig. 2c, d). Briefly, the expression level of *Ptprc* was used to determine the immune cells, with T cells expressing *Cd3g*, *Cd3e*, macrophages expressing higher levels of *Cd68*, *Pf4* and *Adgre1*, and neutrophils displaying significantly elevated *Camp* and *Ngp* levels. In addition, *Reg3g*, *Gap43*, *Omp*, *Gng8, Gng18, Krt14 and Krt5* were applied to distinguish specific cell types in the olfactory membranes, including sustantacular cells, immature olfactory sensing neurons (iOSNs), mature olfactory sensing neurons (mOSNs) and horizontal basal cells (HBCs). To determine the mesenchymal sources, the expression levels of *Col1a1*, *Acta2* and *Prrx1* were used, while osteolineage cells (OLCs) were determined by osteogenic markers, such as *Alpl*, *Runx2* and *Sp7*. The positive expression of *Acan* and *Sox9* represents chondrocytes, and higher levels of *Eng* and *Nes* represent pericytes. Neural progenitor cells (*Ngfr*, *Sox10*) and endothelial cells (*Cdh5*, *Emcn* and *Pecam1*) were also discovered in MSFL-induced bone formation (Supplementary information, Fig. S2e, f). The OLCs were virtually absent, accounting for only 0.05% of the total mNM cells under steady-state condition. However, after MSFL surgery, the proportion of OLCs started to soar and peaked on day 6 (62.4%). Then OLCs gradually declined to 35.7% on day 14 (Fig. 2e), which was consistent with the time course and WGCNA analyses.

After merging OLCs of all time points, 19,723 cells were obtained for subgroup classification and further divided into 9 clusters based on the abundance of osteogenic genes (*Alpl, Sp7, Col1a2, Runx2, Bglap, and Ibsp*) (Fig. 2f, g; Supplementary information, Fig. S3a, b). Pseudotime and the RNA velocity analyses displayed a similar osteogenic differentiation trajectory, originating from the cluster of osteoprogenitor-1 (Op-1) toward the clusters of mature osteoblast (mature ob) and osteocyte (Oc) (Fig. 2h–j).

Interestingly, *Krt14*, a classic epithelial marker,^36–38^ was identified to be Op-1-specific after calculating differential expression genes (DEGs) by comparing Op-1 to the rest of the OLCs (Log_2_FC = 1.218, *P* = 9.9e–56) (Fig. 2k, l). Moreover, the pseudotime differentiation trajectory (Fig. 2r) and amplified RNA velocity (Fig. 2q) of OLCs showed that most *Krt14*^*+*^ OLCs were located in the upstream of the differentiation trajectory of OLCs, indicating that despite possessing epithelial characteristics, these *Krt14*^*+*^ OLCs still possessed a strong osteogenic differentiation capacity. Then we traced the expression of *Krt14* back to total cells. The expression distribution diagram of the whole data set showed that *Krt14* was only expressed in the clusters of HBC and Op-1 (Fig. 2m–p).

To sum up, the single-cell atlas identified a subcluster of *Krt14*^*+*^ osteoprogenitor cells which robustly give rise to the formation of osteogenic niche, despite the epithelial signature at the transcriptional level.

### *Krt14*^*+*^*Ctsk*^*+*^ cells with combined epithelial and mesenchymal characteristics

**Fig. 3:**
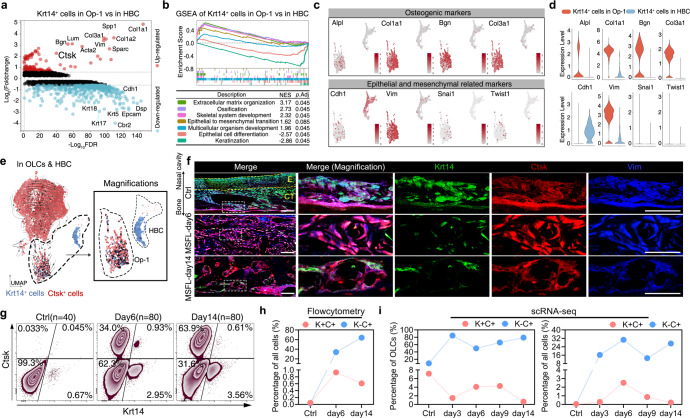
Both epithelial and mesenchymal characteristics presented by *Krt14*^*+*^*Ctsk*^*+*^ cells. **a** Volcano plots showing DEGs of *Krt14*^*+*^ Op-1 versus *Krt14*^*+*^ HBC (cutoff: |Log_2_FC| > 0.58, FDR < 0.1); upregulated genes (red); downregulated genes (blue). **b** GSEA analysis presenting enriched transcriptional programs in *Krt14*^*+*^ Op-1 versus those in *Krt14*^*+*^ HBC; color coded by each program. **c** Expression of osteogenic, epithelial and mesenchymal markers visualized on UMAP projection of Op-1 and HBC; color coded by the expression level of each gene. **d** Violin plots generated from **c**; showing log-normalized expression values of the indicated genes. **e** Distribution of *Krt14*^*+*^ cells (blue) and *Ctsk*^*+*^ cells (red) visualized on the UMAP projection of OLCs and HBCs; color coded by the expression level of each gene. The area circled by the dotted line is magnified on the right. **f** Representative confocal images of nose sections from wild-type mice on Ctrl, days 6, and 14 post-surgery groups. Images in the white dotted boxes were shown in the right part of each panel. Merged and single-channel images of Krt14 (green), Ctsk (red), and Vim (blue) staining were shown. *n* = 3 mice per condition from 3 independent experiments. Scale bar, 50 μm. The yellow dotted lines indicate the boundaries of anatomical landmarks. E, epithelium; CT, connective tissue. **g** Flow cytometry analysis of cells stained by Krt14 and Ctsk. *n* = 40 from the Ctrl group; *n* = 80 for groups of days 6 and 14 post-MSFL. The proportion of each cell type is labeled aside. One independent experiment was carried out. **h** Dot-line plot generated from **f**; *Krt14*^*+*^*Ctsk*^*+*^ cell (red); *Krt14*^*–*^*Ctsk*^*+*^ cell (blue). **i** Dot-line plots showing the variation of *Krt14*^*+*^*Ctsk*^*+*^ cell (red) and *Krt14*^*–*^*Ctsk*^*+*^ cell (blue) proportion at all time points generated from scRNA-seq analysis; the proportion of each cell type in OLCs (left) and in all cells (right) is shown.

To distinguish the transcriptional differences between clusters of *Krt14*^*+*^ Op1 and *Krt14*^*+*^ HBC, the analysis of DEGs was performed. According to the volcano plot, *Krt14*^*+*^ Op-1 showed higher expression of osteogenesis-related genes (*Ctsk, Col1a1, Col1a2, Sparc, Spp1*, and *Col3a1*) and significantly lower epithelial markers (*Cdh1, DSP, Krt15*, and *Epcam*) as compared to *Krt14*^*+*^ HBC (Fig. 3a). By gene set enrichment analysis (GSEA), *Krt14*^*+*^ Op-1 exhibited higher enrichment levels on extracellular matrix organization and mineralization, while *Krt14*^*+*^ HBC mainly displayed epithelial characteristics such as keratinization (Fig. 3b; Supplementary information, Table S1). The gene expression profiles and violin plots consistently indicated that *Krt14*^*+*^ Op-1 expressed more osteogenic genes and fewer epithelial genes as compared to *Krt14*^*+*^ HBC (Fig. 3c, d). The above results were consistent with previous studies, showing that HBCs were the olfactory epithelium-specific stem cells,^39,40^ which have never been reported to be osteogenic. Based on the different biological behaviors presented by *Krt14*^*+*^ Op-1 and *Krt14*^*+*^ HBC, we used the second marker from significantly elevated DEGs, *Ctsk*, which had been shown to be a bona fide marker of periosteum stem cells,^16^ to distinguish *Krt14*^*+*^ Op-1 from *Krt14*^*+*^ HBC. By labeling the *Krt14*^*+*^ and *Ctsk*^*+*^ cells on UMAP projection of OLCs and HBCs, the *Krt14*^*+*^*Ctsk*^*+*^ cells were almost exclusively distributed in Op-1, as shown in Fig. 3e.

The presence and localization of *Krt14*^*+*^*Ctsk*^*+*^ cells were confirmed by immunofluorescence labeling. In mNM, *Krt14* expression gradually decreased along the nasal cavity to bone axis, while *Ctsk* expression increased. In addition, Vimentin (Vim), a mesenchymal marker,^41^ was co-labeled with Ctsk. *Krt14*^*–*^*Ctsk*^*+*^ cells were scattered in the bottom layer next to the bone floor. A small amount of *Krt14*^*+*^*Ctsk*^*+*^ cells were located in the middle layer under homeostasis (Fig. 3f). Flow cytometry results showed that the proportion of *Krt14*^*–*^*Ctsk*^*+*^ osteogenic cells, which resemble periosteum mesenchymal cells, was ~0.033%, and that the proportion of *Krt14*^*+*^*Ctsk*^*+*^ cells was 0.045% in the preoperative mNM (Fig. 3g, h).

Six days after MSFL, we observed that more *Krt14*^*+*^ cells became co-labeled with Ctsk and vimentin in the regeneration area (Fig. 3f). Flow cytometry also revealed that the proportion of *Krt14*^*+*^*Ctsk*^*+*^ cells (0.93%) peaked on day 6 (Fig. 3g, h), which was consistent with the peak appearance of the Op-1 population in scRNA-seq (Fig. 3i). By day 14, the number of *Krt14*^*+*^*Ctsk*^*+*^ cells distributed on the bone surface decreased, especially in the mature bone region (Fig. 3f). Flow cytometry also showed that the proportion of *Krt14*^*+*^*Ctsk*^*+*^ cells declined to 0.61% (Fig. 3g, h), and scRNA-seq displayed a similar tendency (Fig. 3i).

**Fig. 4:**
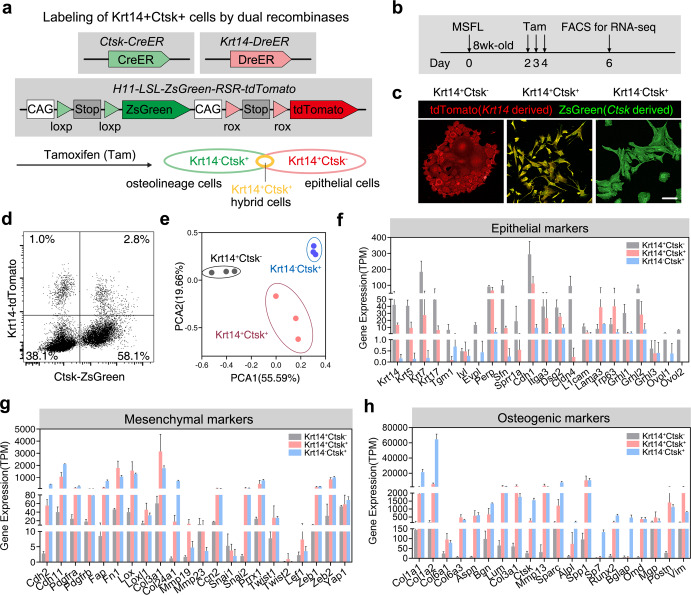
Transcriptomic features of *Krt14*^*+*^*Ctsk*^*+*^ cells. **a** Illustration of dual recombinases-mediated lineage tracing strategy by generating *Krt14*^*DreER*^*;Ctsk*^*CreER*^*;H11*^*RSR-tdTomato-LSL-ZsGreen*^ mouse. TAM treatment resulted in ZsGreen-labeled *Krt14*^–^*Ctsk*^*+*^ cells (green), tdTomato-labeled *Krt14*^*+*^*Ctsk*^–^ cells (red), and ZsGreen and tdTomato double-labeled *Krt14*^*+*^*Ctsk*^*+*^ cells (yellow). **b** Workflow of TAM treatment, FACS, and subsequent RNA-seq. **c** Representative confocal images of FACS-sorted *Krt14*^*+*^*Ctsk*^–^ cells (red), *Krt14*^*+*^*Ctsk*^*+*^ cells (yellow), and *Krt14*^–^*Ctsk*^*+*^ cells (green) cultured on cell climbing films for 48 h. Scale bar, 25 μm. *n* = 3 from 3 independent experiments. **d** Proportions of three types of cells sorted by FACS. *n* = 20 from one independent experiment. **e** PCA indicating the hierarchical clustering and variations of transcriptomes among *Krt14*^*+*^*Ctsk*^–^, *Krt14*^*+*^*Ctsk*^*+*^, and *Krt14*^–^*Ctsk*^*+*^ cells. **f**–**h** TPM analysis of epithelial-, mesenchymal-, and osteogenic-related marker genes in FACS-sorted subpopulations defined by RNA-seq, respectively. *n* = 3; means ± SD.

To further verify whether *Krt14*^*+*^*Ctsk*^*+*^ cells from the regenerated tissue possessed bona fide epithelial and mesenchymal features, we applied a double recombinases-mediated lineage tracing system to label *Krt14*^*+*^*Ctsk*^*+*^ cells in vivo. *Krt14*^*dreER*^*;Ctsk*^*creER*^*;H11*^*LSL-ZsGreen-RSR-tdTomato*^ mice were generated by crossing *Krt14*^*dreER*^, *Ctsk*^*creER*^, and *H11*^*RSR-tdTomato-LSL-ZsGreen*^ mice (Fig. 4a).

Tamoxifen (TAM) was administered continuously for 3 days from the second day after MSFL (Fig. 4b). After TAM treatment, both Cre-loxP and Dre-roxP recombination were induced simultaneously, thus resulting in ZsGreen-labeled *Krt14*^*–*^*Ctsk*^*+*^ cells (green), tdTomato-labeled *Krt14*^*+*^*Ctsk*^*–*^ cells (red), and ZsGreen and tdTomato double-labeled *Krt14*^*+*^*Ctsk*^*+*^ cells (yellow) (Fig. 4c). Three types of cells were collected on day 6 by FACS and cultured for RNA-seq (Fig. 4d).

Hierarchical clustering by principal component analysis (PCA) showed that the *Krt14*^*+*^*Ctsk*^*+*^ cells were more similar to *Krt14*^*–*^*Ctsk*^*+*^ osteogenic cells (Fig. 4e). The results suggested that the transcription levels of epithelial, mesenchymal, and osteogenic feature genes were substantially different between *Krt14*^*+*^*Ctsk*^*–*^ epithelial and *Krt14*^*–*^*Ctsk*^*+*^ osteogenic cells (Fig. 4f–h), which was consistent with the PCA analysis (Fig. 4e). Furthermore, expression levels of epithelial markers were higher in *Krt14*^*+*^*Ctsk*^*+*^ cells than those in *Krt14*^*–*^*Ctsk*^*+*^ osteogenic cells, indicating a more prominent epithelial characteristic of *Krt14*^*+*^*Ctsk*^*+*^ cells (Fig. 4f). Correspondingly, these cells demonstrated a stronger osteogenic feature than *Krt14*^*+*^*Ctsk*^*–*^ epithelial cells but a weaker osteogenic feature than *Krt14*^*–*^*Ctsk*^*+*^ cells (Fig. 4h). Meanwhile, the mesenchymal-related gene expression profiling suggested that *Krt14*^*+*^*Ctsk*^*+*^ cells display more mesenchymal characteristics than *Krt14*^*+*^*Ctsk*^*–*^ cells (Fig. 4g).

### *Krt14*^*+*^*Ctsk*^*+*^ cells contribute to maxillofacial bone homeostasis and MSFL-induced osteogenesis

**Fig. 5:**
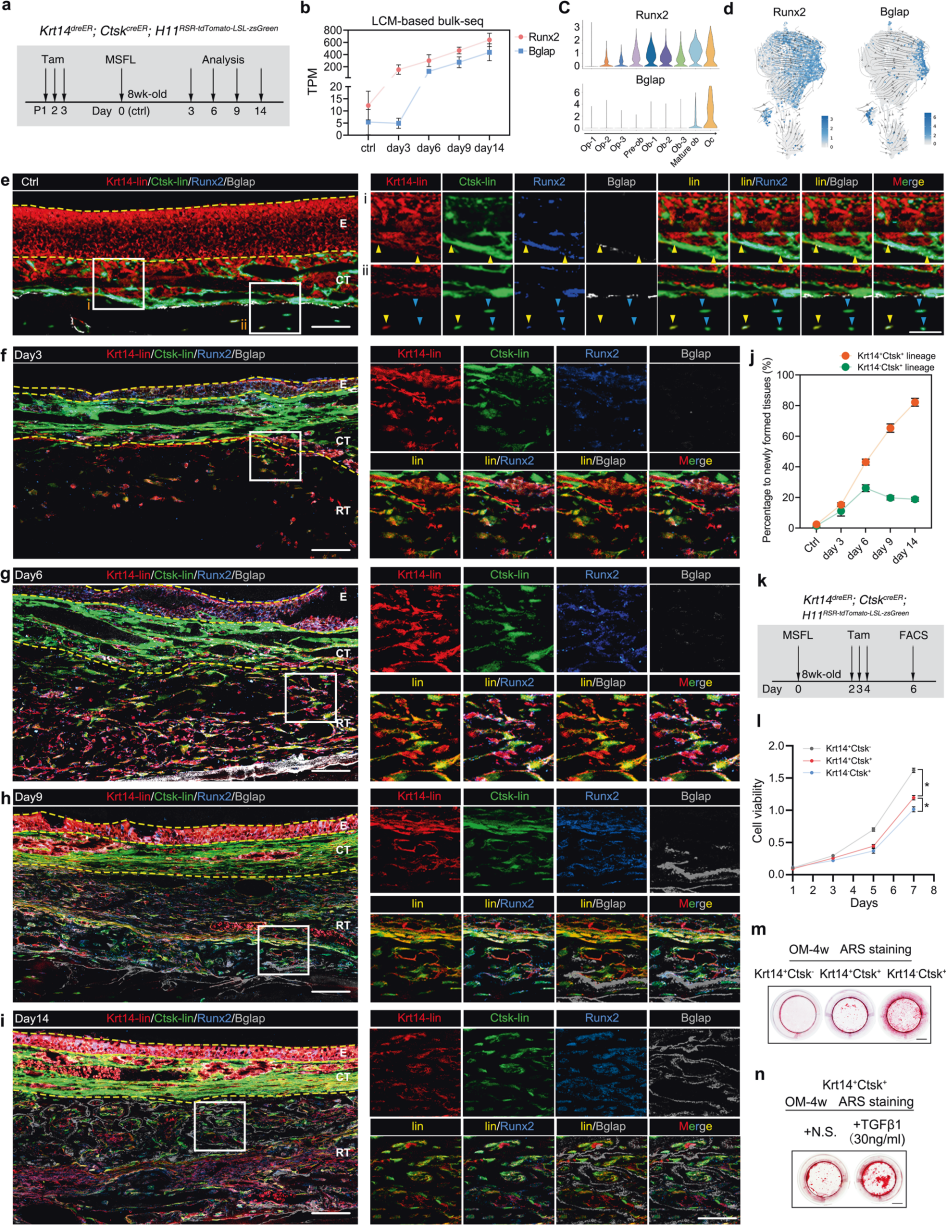
*Krt14*^*+*^*Ctsk*^*+*^ cells contribute to MSFL-induced bone regeneration. **a** TAM treatment strategy with *Krt14*^*DreER*^*;Ctsk*^*CreER*^*;H11*^*RSR-tdTomato-LSL-ZsGreen*^ mice to perform lineage tracing. TAM was injected for 3 continuous days from the P1. **b** TPM of *Runx2* and *Bglap* indicated by LCM-based bulk RNA-seq analysis at different time points. *n* = 3; means ± SD. **c** Violin plots showing the distribution and log-normalized expression values of *Runx2* and *Bglap* in 9 subclusters of OLCs. **d** Expression of *Runx2* (left) and *Bglap* (right) visualized on UMAP projection of OLCs with RNA velocity; color coded by the expression level of each gene. **e**–**i** Representative confocal images of sample sections from Ctrl (**e**), day 3 (**f**), day 6 (**g**), day 9 (**h**), and day 14 (**i**). Area in the box is magnified on the right of each panel. Merged and single-channel images of Krt14 (red), Ctsk (green), Runx2 (blue), and Bglap (gray) were shown. Yellow arrowheads indicate *Krt14*^*+*^*Ctsk*^*+*^ cells; blue arrowheads indicate *Krt14*^–^*Ctsk*^*+*^ cells. Yellow dotted lines indicate the boundaries of anatomical landmarks. E epithelium, CT connective tissue, RT regenerative tissue. For **e**–**i**, scale bar, 50 μm; lin, lineage; *n* = 3 from 3 independent experiments. **j** Percentage variation of *Krt14*^*+*^*Ctsk*^*+*^ cells and *Krt14*^–^*Ctsk*^*+*^ cells in the newly formed tissue generated from **e**–**i**; means ± SD. **k** TAM treatment strategy with *Krt14*^*DreER*^*;Ctsk*^*CreER*^*;H11*^*RSR-tdTomato-LSL-ZsGreen*^ mice for the subsequent FACS sorting. TAM was injected for 3 continuous days from the second postoperative day. **l** CCK-8 assay of FACS-sorted *Krt14*^*+*^*Ctsk*^–^ cells, *Krt14*^*+*^*Ctsk*^*+*^ cells, and *Krt14*^–^*Ctsk*^*+*^ cells. *n* = 5, *P < 0.05, one-way ANOVA followed by Dunnett’s multiple comparisons test. **m** Representative ARS images of *Krt14*^*+*^*Ctsk*^–^ cells, *Krt14*^*+*^*Ctsk*^*+*^ cells, and *Krt14*^–^*Ctsk*^*+*^ cells after culturing in osteogenic medium (OM) for 4 weeks, indicating the osteogenic capacity and extracellular mineralization level of each type of cells. Scale bar, 2 mm; *n* = 3 from 3 independent experiments. **n** Representative ARS images of *Krt14*^*+*^*Ctsk*^*+*^ cells after culturing in OM supplemented with normal saline (N.S.) or 30 ng/mL TGF-β1 for 4 weeks. Scale bar, 2 mm; *n* = 3 from 3 independent experiments.

To elucidate the in vivo function of *Krt14*^*+*^*Ctsk*^*+*^ cells, dual recombinases-mediated lineage tracing system was applied by generating *Krt14*^*DreER*^*;Ctsk*^*CreER*^*;H11*^*LSL-ZsGreen-RSR-tdTomato*^ mouse. TAM was administered for three continuous days from postnatal day 1 (P1) and MSFL surgery was performed on week 8 (Fig. 5a).

Elevated region in sectioned tissue samples from pre-MSFL (Ctrl) and days 3, 6, 9, and 14 post-MSFL were collected for LCM-based bulk RNA-seq. The expression of the classic osteogenic transcription factor Runx2 started to soar soon after MSFL, while the growth rate of *Runx2* TPM slowed down since postoperative day 3 (Fig. 5b). Variation of *Runx2* expression and RNA velocity calculated from scRNA-seq data also showed that *Runx2* was expressed at the early stage of osteogenic differentiation (Fig. 5c, d). *Bglap*, also known as the protein-coding gene of osteocalcin, was mainly expressed in Oc and mature Ob clusters, indicating a later expression pattern compared to *Runx2*. The TPM of *Bglap* also displayed a delayed rise (Fig. 5b–d). Therefore, *Runx2* and *Bglap* were applied to depict the maturation process of *Krt14*^*+*^*Ctsk*^*+*^ cells in both maxilla bone homeostasis and MSFL-induced bone regeneration.

Herein, sections from lineage tracing mice were co-stained with Runx2 (blue) and Bglap (gray). Under steady state, *Krt14*^*+*^*Ctsk*^*+*^ cells (yellow) were scattered in the middle and bottom layers of mNM. Additionally, some *Krt14*^*+*^*Ctsk*^*+*^ cells lining on the interface between mNM and bone were Runx2-positive, suggesting an osteogenic differentiation potential. Interestingly, some *Krt14*^*+*^*Ctsk*^*+*^ cells could be found within the bone tissue, suggesting that *Krt14*^*+*^*Ctsk*^*+*^ cells might contribute to the bone turnover under homeostasis (arrows, Fig. 4e). On days 3 and 6, the layer below the split line displayed robust proliferation. More *Krt14*^*+*^*Ctsk*^*+*^ cells appeared with stronger Runx2 signals, suggesting that the *Krt14*^*+*^*Ctsk*^*+*^ cells were propelling early-stage bone formation by osteogenic differentiation (Fig. 5f, g). On day 9, the elevated zone become thicker. More *Krt14*^*+*^*Ctsk*^*+*^*Runx2*^*+*^ cells were located in the newly-formed bone. Moreover, some of the *Krt14*^*+*^*Ctsk*^*+*^*Runx2*^*+*^ cells were *Bglap*^low^, and the number of *Krt14*^*+*^*Ctsk*^*+*^*Runx2*^*+*^*Bglap*^*+*^ cells increased on day 14, suggesting that the *Krt14*^*+*^*Ctsk*^*+*^ cells could further push forward the late-stage bone formation (Fig. 5h, i). The statistics of the lineage proportions depicted that the majority of the osteogenesis in the elevated region was contributed by *Krt14*^*+*^*Ctsk*^*+*^ lineage (Fig. 5j).

The functional verifications of *Krt14*^*+*^*Ctsk*^*+*^ lineage cells were also carried out in transalveolar MSFL murine model, with the application of *Rosa26*^*LSL-tdTomato*^*;Krt14*^*CreER*^ reporter line. With TAM administration on P1 to P3, some *Krt14*^*+*^ lineage cells were observed to participate in the homeostatic bone turnover and co-stained with Ctsk in the control group (Supplementary information, Fig. S4a). On days 3, 6, and 9, the signals of *Krt14*^*+*^ lineages, co-stained with Ctsk, were widely distributed in the regenerative tissues, indicating a similar osteogenic role as they played in the transnasal MSFL model (Supplementary information, Fig. S4b–d).

Besides in vivo lineage tracing, the in vitro cell viability and osteogenic assays were completed by sorting in vivo labeled *Krt14*^*+*^*Ctsk*^*–*^, *Krt14*^*+*^*Ctsk*^*+*^ and *Krt14*^*–*^*Ctsk*^*+*^ cells from newly formed tissues after MSFL with TAM treatment (Fig. 5k). When compared with *Krt14*^*–*^*Ctsk*^*+*^ cells, the *Krt14*^*+*^*Ctsk*^*+*^ cells exhibited higher proliferation rate (Fig. 5l), along with relatively weak osteogenic capacities, as indicated by the formed calcium nodes marked by Alizarin Red S (ARS) staining, which suggested that the epithelial characteristics remained in *Krt14*^*+*^*Ctsk*^*+*^ cells (Fig. 5m). With the treatment of TGF-β1, the osteogenic outcome of *Krt14*^*+*^*Ctsk*^*+*^ cells was significantly improved (Fig. 5n).

### Maturation of *Krt14*^*+*^*Ctsk*^*+*^ cells undergoing conversion to pure *Krt14*^*–*^*Ctsk*^*+*^ osteolineage cells

In view of the differentiation trajectory from *Krt14*^*+*^*Ctsk*^*+*^ cells to *Krt14*^*–*^*Ctsk*^*+*^ cells implicated in scRNA-seq (Fig. 3e) and the increasing proportion of *Krt14*^*+*^*Ctsk*^*+*^ cells contributing to osteogenesis (Fig. 5j), we hypothesized a probable conversion from *Krt14*^*+*^*Ctsk*^*+*^ cells to *Krt14*^*–*^*Ctsk*^*+*^ osteolineage cells during MSFL-induced osteogenesis.

**Fig. 6:**
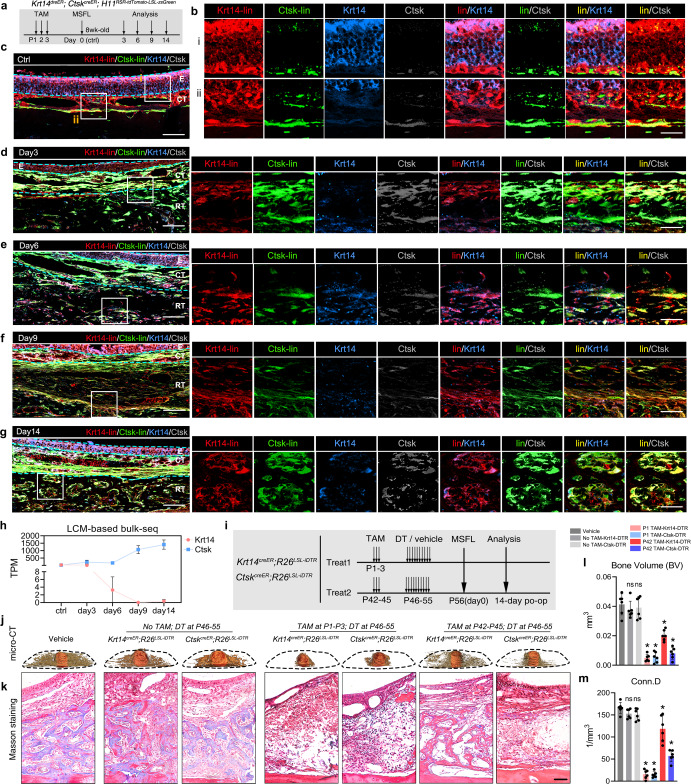
*Krt14*^*+*^*Ctsk*^*+*^ cells convert to *Krt14*^–^*Ctsk*^*+*^ osteogenic descendants. **a** TAM treatment strategy with *Krt14*^*DreER*^*;Ctsk*^*CreER*^*;H11*^*RSR-tdTomato-LSL-ZsGreen*^ mice to perform lineage tracing. TAM was injected for 3 continuous days from the P1. **b**–**g** Representative confocal images of sample sections from Ctrl (**b**, **c**), day 3 (**d**), day 6 (**e**), day 9 (**f**), and day 14 (**g**) showing expression of Krt14 and Ctsk by lineage tracing (lin) combined with immunofluorescence staining. Area in the box is magnified on the right of each panel. Merged and single-channel images of Krt14-lin (red), Ctsk-lin (green), Krt14 (blue), and Ctsk (gray) were shown. Cyan dotted lines indicate the boundaries of anatomical landmarks. E epithelium, CT connective tissue, RT regenerative tissue. For **b**–**g**, scale bar, 50 μm; “lin” in red indicating Krt14-lin; “lin” in green indicating Ctsk-lin; “lin” in yellow indicating Krt14-lin merged with Ctsk-lin; *n* = 3 from 3 independent experiments. **h** TPM of *Krt14* or *Ctsk* indicated by LCM-based bulk RNA-seq analysis at different time points. *n* = 3; means ± SD. **i** Strategy for selective cell depletion with *Krt14*^*CreER*^*;R26*^*LSL-iDTR*^ or *Ctsk*^*CreER*^*;R26*^*LSL-iDTR*^ mice. For treatment 1, TAM was injected for 3 continuous days in P1–P3. For treatment 2, TAM was injected for 3 continuous days in P42–P45. In both treatments, DT or vehicle was injected for 10 days before MSFL to kill *Krt14*^*+*^ cells or *Ctsk*^*+*^ cells selectively. **j** Representative images of 3D reconstruction; orange, implant; brown, bone. *n* = 6 mice from 6 independent experiments. **k** Representative masson staining images representing the changes in histological microstructures in augmented regions. *n* = 6 mice per treatment from 6 independent experiments. Scale bar, 50 μm. **l**, **m** Evaluation of the absolute bone volume (BV) (**l**) and connective density (Conn.D.) (**m**) of lifted regions. Data are presented as the means ± SD of 6 mice per treatment from 6 independent experiments. *P < 0.05, one-way ANOVA followed by Dunnett’s multiple comparisons test was used, with the vehicle group set as control.

Herein, the sections from double lineage tracing mice, treated with the same strategy mentioned previously (Figs. 5a, 6a), were co-stained with Krt14 (blue) and Ctsk (gray) antibodies. Under homeostasis, signals of Krt14 proteins (blue) were almost colocalized with its lineage signals (red), with the latter providing stronger signals in the basal layers of epithelium and gland cells. Signals of Ctsk proteins (gray) were mainly localized on the bone surface, while positive signals could also be found in some epithelium structures in Ctsk lineage (green).

From days 3 to 14, the expression level of Krt14 protein in *Krt14*^*+*^*Ctsk*^*+*^ lineage cells declined over time, while the Ctsk protein level (gray) increased gradually in *Krt14*^*+*^*Ctsk*^*+*^ lineage cells (Fig. 6c–g), which implied that the *Krt14*^*+*^*Ctsk*^*+*^ lineage cells underwent a conversion from *Krt14*^*+*^*Ctsk*^*+*^ to *Krt14*^*–*^*Ctsk*^*+*^ during the development and maturation of newly formed bone tissues. This histological-level observation was also validated by LCM-based bulk RNA-seq of the elevated region, which displayed the increasing *Ctsk* and decreasing *Krt14* mRNA levels over time (Fig. 6h). Similar observations could be made in vitro. The declined signals of Krt14 and E-cadherin, along with strengthened Alpl signal were observed in FACS-sorted *Krt14*^*+*^*Ctsk*^*+*^ cells during in vitro osteogenic induction, indicating the fading epithelial characteristics and uprising osteogenic features (Supplementary information, Fig. S5a).

To further verify the lineage tracing results, we performed lineage elimination studies. *Rosa26*^*LSL-iDTR-GFP*^ mice were mated with *Ctsk*^*CreER*^ or *Krt14*^*CreER*^ mice to obtain *Ctsk*^*CreER*^*;R26*^*LSL-iDTR*^ and *Krt14*^*CreER*^*;R26*^*LSL-iDTR*^ mice. TAM was injected at P1–P3 or P42–P44. Diphtheria toxin (DT) was injected locally at P46–P55, and mouse MSFL was performed at the age of 8 weeks (Fig. 6i). In addition to the treatment groups, the controls of both lines with DT but not TAM administration were also included. On day 14 post MSFL, there was no bone regeneration in the presumptive regeneration region of *Krt14*^*CreER*^*;R26*^*LSL-iDTR*^ mice when TAM was injected at P1–P3. Only a few fibroid cells were found near the bone surface. Moreover, the respiratory mucosa of this group was drastically changed, and the epithelial cells were almost completely apoptotic. Similarly, no obvious MSFL-induced bone formation was observed in *Ctsk*^*CreER*^*;R26*^*LSL-iDTR*^ mice. Only a small amount of osteoid was found near the bone surface in this group. Yet, the structure of the mucosal surface and interlayer appeared to be intact, which was expected. When TAM was injected at P42–P44, after MSFL, the mucosal surface and intermediate layer of *Krt14*^*CreER*^*;R26*^*LSL-iDTR*^ mice underwent atrophy. The bone regeneration volume in the regeneration area was significantly reduced compared with that in the vehicle group. On the other hand, in *Ctsk*^*iDTR*^ mice, MSFL-induced osteogenesis appeared to be severely impaired even if TAM was injected at P42–P44 (Fig. 6k). These results are consistent with those of the Micro-CT analysis (Fig. 6j, l, m).

### Characterization of *Krt14*^*+*^*Ctsk*^*+*^ cells in hSM

**Fig. 7:**
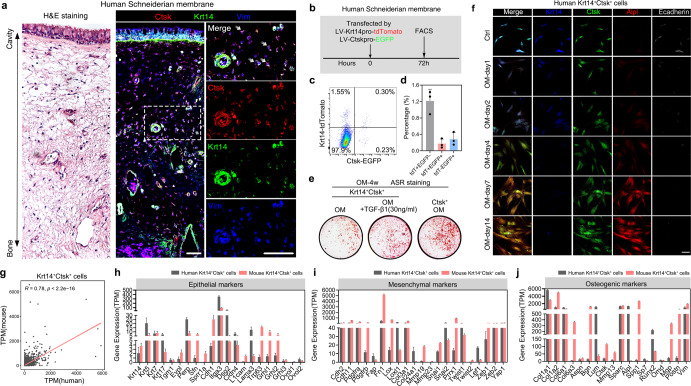
*Krt14*^*+*^*Ctsk*^*+*^ cells in hSM. **a** Representative H&E staining image of hSM (left) and confocal images of hSM under homeostatic condition (right). Merged and single-channel images of Ctsk (red), Krt14 (green), and Vim (blue) are shown in each panel. *n* = 3 from 3 different experiments; area in the white dotted box is magnified on the right; scale bar, 50 μm. **b** Illustration showing the strategy for *Krt14*^*+*^*Ctsk*^*+*^ cell labeling in hSM by LV-*Krt14*pro-tdTomato + LV-*Ctsk*pro-EGFP, and for tdT^+^EGFP^+^ (representing *Krt14*^*+*^*Ctsk*^*+*^ cells), tdT^+^EGFP^–^ (representing *Krt14*^+^*Ctsk*^–^ cells) and tdT^–^EGFP^+^ (representing *Krt14*^–^*Ctsk*^*+*^ cells) cell sorting by FASC after 72 h of infection. **c** Proportion of FACS-sorted cells. **d** Histogram generated from **c**. Data are presented by means ± SD; *n* = 3. **e** Representative ARS images showing osteogenic differentiation capacities of *Krt14*^*+*^*Ctsk*^*+*^ cells cultured in OM versus *Krt14*^*+*^*Ctsk*^*+*^ cells in OM + TGF-β1 versus *Krt14*^–^*Ctsk*^*+*^ cells in OM after 4 weeks. **f** Representative confocal images of human *Krt14*^*+*^*Ctsk*^*+*^ cells cultured in OM at different time points. Merged and single-channel images of Ctsk (green), Krt14 (blue), Cdh1 (gray) and Alpl (red) are shown in each panel. Scale bar, 25 μm. *n* = 3 from 3 independent experiments. **g** Correlation analysis of *Krt14*^*+*^*Ctsk*^*+*^ cells from hSM and mNM at transcriptomic level. TPM reads of genes were analyzed by Spearman correlation analysis. *R* = 0.78, *P* < 2.2e–16. **h**–**j** Comparison of TPM of epithelial (**h**), mesenchymal (**i**) and osteogenic (**j**) specific markers in *Krt14*^*+*^*Ctsk*^*+*^ cells from hSM and mNM. Error bars indicate SEM.

To verify whether *Krt14*^*+*^*Ctsk*^*+*^ cells also exist in hSM, multiple immunofluorescence staining was performed. The *Krt14*^*+*^*Ctsk*^*+*^ cells were scattered in the loose connective tissue layer, which colocalized with Vim signals (Fig. 7a). By dissecting the membrane and labeling the *Krt14*^*+*^*Ctsk*^*+*^ cells by lentivirus-mediated expression of fluorescent proteins (tdTomato and EGFP) driven by respective promoters, we acquired *Krt14*^*+*^*Ctsk*^*+*^ and *Krt14*^*–*^*Ctsk*^*+*^ cells by FACS (Fig. 7b). In hSM, the proportions of *Krt14*^*+*^*Ctsk*^*+*^ and *Krt14*^*–*^*Ctsk*^*+*^ cells reached 0.30% and 0.23%, respectively (Fig. 7c, d), and both of the cells display osteogenic capacities (Fig. 7e). By adding TGF-β1, the osteogenic potential of *Krt14*^*+*^*Ctsk*^*+*^ cells significantly surged, as indicated by ARS staining (Fig. 7e). In in vitro osteogenic assays, the gradual fading epithelial characteristics and uprising osteogenic features of *Krt14*^*+*^*Ctsk*^*+*^ cells were observed, as indicated by co-staining of Krt14, Ctsk, Alpl and E-cadherin (Fig. 7f).

**Fig. 8:**
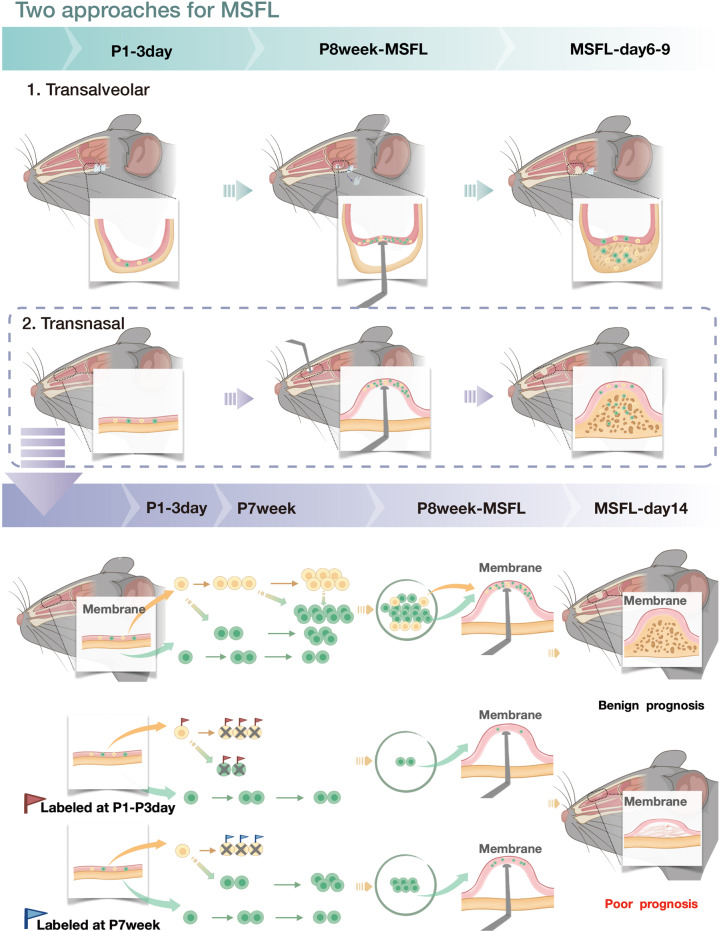
A schematic diagram depicting *Krt14*^*+*^*Ctsk*^*+*^ cell transition and the role of *Krt14*^*+*^*Ctsk*^*+*^ cells in bone regeneration. The upper part of the diagram depicts the two approaches for MSFL and the transnasal approach is used for most experiments. The middle part illustrates the relative proportions of *Krt14*^*+*^*Ctsk*^*+*^ (yellow) and *Krt14*^–^*Ctsk*^*+*^ (green) cells and their transitions after MSFL. The lower part demonstrates the labeling strategy for DT-based cell depletion and the effect on the outcomes of MSFL-induced bone regeneration. Red flags, labeled by TAM at postnatal days 1–3; blue flags, labeled by TAM at postnatal week 7; cross, killed by DT.

To verify the similarities of the cells, bulk RNA-seq was carried out. After quality control, normalization, and matching homologous genes, TPM reads of protein-coding genes were compared by Spearman correlation analysis. The results showed a significant correlation between the transcriptomes of *Krt14*^*+*^*Ctsk*^*+*^ cells from human and mouse (*R* = 0.78, *P* < 2.2e–16) (Fig. 7g). In addition, by evaluating the TPM of epithelial, mesenchymal and osteogenic markers between the cells of two sources, we found that the human being-derived *Krt14*^*+*^*Ctsk*^*+*^ cells exhibited less epithelial characteristics but a stronger osteogenic potential (Fig. 7h–j). The schematic diagram of this study was presented in Fig. 8.

### Characterization of the origin of *Krt14*^*+*^*Ctsk*^*+*^ cells during embryonic development

To explore the developmental origin of *Krt14*^*+*^*Ctsk*^*+*^ cells, *Krt14*^*CreER*^*;R26*^*LSL-tdTomato*^ mice were injected with TAM at embryonic day 10.5 (E10.5), and samples were collected at E12.5, E15.5, and P1 (Supplementary information, Fig. S6a). The distribution of *Krt14*^*+*^ epithelial lineage cells in the pre-respiratory mucosa was analyzed. In all three groups of different ages, most *Krt14*^*+*^ cells were co-labeled with the Ctsk signal, and could be found in the regions about to form the SM, and few cells only expressed Ctsk (Supplementary information, Fig. S6b–e).

Multi-color immunohistochemistry analysis of wild-type embryonic mice without TAM treatment showed that the arising of Krt14 signal (E9.5) was ahead of the appearance of Ctsk signal, which was mainly distributed in the surface ectoderm and the cells nearby (Supplementary information, Fig. S6f). On E10.5, the Ctsk signal started to appear and colocalized with both Krt14 and E-cadherin signals. From E11.5 to 13.5, the pre-respiratory mucosa was almost colocalized with the signals of Krt14, Ctsk and E-cadherin, with some *Krt14*^*+*^*Ctsk*^*+*^*Cdh1*^*+*^ cells located in the mesenchyme nearby. Similar to that of multi-color staining, the Ctsk signal also appeared in the early epithelial layer, and was located in the mesenchyme nearby in the lineage tracing group (Supplementary information, Fig. S6b).

In E15.5 and P1, the SM-like structure appeared, with *Krt14*^*+*^*Ctsk*^*+*^ cells mainly located at the mesenchyme nearby and limited *Krt14*^*+*^*Ctsk*^*+*^*Cdh1*^*+*^ cells residing in the epithelial layer, which was indicated by both wild-type mice and lineage tracing experiments (Supplementary information, Fig. S6b, k, l).

To evaluate the transcriptomic features of *Krt14*^*+*^*Ctsk*^*+*^ cells in the embryonic period, we acquired *Krt14*^*+*^*Ctsk*^*+*^ cells of E15.5 from SM-like tissues of double lineage tracing mice by FACS (Supplementary information, Fig. S6m). In the tissue, the proportions of *Krt14*^*+*^*Ctsk*^*+*^ and *Krt14*^*–*^*Ctsk*^*+*^ cells reached 5.42% and 1.05%, respectively (Supplementary information, Fig. S6n, o). To verify the similarities of the cells, bulk RNA-seq was carried out. After quality control and normalization, TPM reads of protein-coding genes were compared by Spearman correlation analysis. The results showed a significant correlation between the transcriptomes of *Krt14*^*+*^*Ctsk*^*+*^ cells from embryonic and adult mice (*R* = 0.95, *P* < 2.2e–16) (Supplementary information, Fig. S6p).

## Discussion

Bone repair and regeneration rely on the activation of osteoprogenitor cells, which are located in the epiphysis marrow,^9^ growth plate,^14,15^ periosteum^16–18^ and cranial suture.^16,22,24,25,42^ Osteoprogenitors mediate bone regeneration by endochondral or intramembranous ossification.^1,6,43,44^ Endochondral ossification is a process starting from the entry of blood vessels and cartilage progenitor cells into the center of the cartilage plate to form the primary ossification center, where osteoblasts, vascular endothelial cells, pericytes, and hematopoietic cells cooperate to deposit bone on the remaining cartilage matrix.^43^ Intramembranous ossification involves the aggregation and direct mineralization of cranial mesenchyme; meanwhile the number of osteoblasts increases as a result of osteoprogenitor differentiation.^5,6^ Notably, the proliferation and differentiation capacity of osteoprogenitors decreases significantly with age^19,20^ or after menopause.^21^

MSFL is a classic surgical procedure for guiding bone regeneration and widely used for bone augmentation in the posterior region of atrophic maxilla, in which bone formation between the sinus floor and SM may easily be induced by lifting the membrane.^31^ Mucous membrane is a membrane structure which consists of epithelium and connective tissue in organisms (mouth, organ, stomach, intestine, and urethra, etc.). The cavity of maxillary sinus is covered by SM, which is the extension of respiratory epithelium, the same with the NM that lines the nasal cavity.^28^ It consists of columnar epithelium and connective tissue containing glands, and is histologically different from periosteum covering bones of the extremities and trunk.^29,30^

By analyzing 1210 clinical MSFL cases, we found that the height of regenerated bone at the sinus floor was independent of age and gender, but was closely related to the elevated height of SM. This type of osteogenesis seems different from the traditional theories of endochondral or intramembranous ossification. To investigate the underlying mechanism related to MSFL-induced bone formation, we established murine MSFL models through the transnasal and transalveolar approaches with mini-implants (0.8 mm in nasal cavity and 0.4 mm in maxillary sinus, in view of the narrow sinus volume) (Fig. 1g). Both models displayed bone formation and the regenerated tissues showed similar structure at all time points. In addition, the mSM and nasal respiratory mucosa also shared similar characteristics in the overall transcriptome. Due to the narrow space in mouse maxillary sinus, the sample volume is scarce for the following scRNA-seq. Thus, the transnasal MSFL model was used for the subsequent analysis.

Although common transcriptomic features were shown between hSM and mNM, some dissimilarities also existed. Histological data indicated that hSM had relatively thin epithelium and thick loose connective tissue, whereas mNM was featured by thicker epithelium and comparatively well-developed glands. Such histological features are well correlated with the respective expression levels of different markers. In detail, hSM shows lower *Cdh1* expression and significantly higher expression levels of endothelial markers. Moreover, the higher *Cd3e* level indicates that more tissue-resident T cells might be present in hSM, indicating the potent antiviral properties of sinus mucosa.^45^ Specifically, the *Krt5*^*+*^ epithelial cells might account for the majority of the horizontal basal cells in hSM, instead of *Krt14*^*+*^ horizontal basal cells in mNM.^46^

To further dissect the cellular composition and lineage hierarchy within heterogeneous cell populations, scRNA-seq of regenerative tissues collected on days 3, 6, 9 and 14 post operation was carried out.^47^ A unique cell subset, primarily located in the initiating cluster of OLCs, was identified to co-express epithelial (*Krt14*) and mesenchymal (*Ctsk*) markers (Fig. 3) and could hardly be divided into heterogeneous subclusters, suggesting their homogeneity. By FACS-based bulk RNA-seq, we further proved that this group of cells showed the characteristics of osteoprogenitor cells and both epithelial and mesenchymal transcript profiles (Fig. 4).

Ctsk is a member of the papain family of cysteine proteases that is highly expressed by activated osteoclasts.^48^ However, Yang et al.^49^ found that Ctsk was not only expressed in osteoclasts but also located in the perichondrial groove of Ranvier. Debnath et al.^16^ identified for the first time the periosteal identity of the *Ctsk*^*+*^ clusters, including subsets of periosteal stem cells, stromal cells and fibroblasts. It is observed in our study that the osteogenic process of MSFL-induced bone regeneration shared some similarities with the approach of intramembranous ossification, with direct differentiation of progenitor cells into osteoblasts and the deposition of osteoid. Given the contribution of *Krt14*^*+*^*Ctsk*^*+*^ lineage in MSFL-induced bone in-growth, it is suggested that the signature functional properties of this lineage might be specialized for intramembranous ossification, which is in line with the previous reports of *Ctsk*^*+*^ cells.

Keratin is the most abundant structural protein in the cytoplasm of epithelial cells. Two-thirds of the keratin genes are found in the cutaneous epithelium, and the family member *Krt14* is mainly expressed in basal cells of epithelial tissues (including the skin, esophagus, salivary glands, small intestine, and mucosa) physiologically^50–52^ and in poorly differentiated tumor cells pathologically, which form well-defined boundary with connective tissue underneath the basement membrane.^53^ The properties of Krt14 suggested that the *Krt14*^*+*^*Ctsk*^*+*^ subset possessed epithelial characteristics. Apart from providing structural supports for cytoskeleton, it was reported that Krt14 regulates the proliferation and differentiation of epithelial cells, which determines their stemness.^54,55^ In our study, the *Krt14*^*+*^*Ctsk*^*+*^ cells exhibited higher proliferation but slightly lower osteogenic capacities when compared with *Krt14*^*–*^*Ctsk*^*+*^ cells. From the evidence provided by both in vivo lineage tracing and in vitro osteogenic differentiation assays, it was observed that the *Krt14*^*+*^*Ctsk*^*+*^ cells differentiated into *Krt14*^*–*^*Ctsk*^*+*^ cells and played robust osteogenic roles; and this process was accelerated and strengthened by administering TGF-β1. This suggested that the expression level of *Krt14* in this subset might not only determine the proliferation and differentiation potentials of cells, but also define the possible heterogeneity of this cluster.

In the present study, we performed dual recombinases-mediated lineage tracing and clarified the localization and fate of *Krt14*^*+*^*Ctsk*^*+*^ lineage cells. Homeostatically, *Krt14*^*+*^*Ctsk*^*+*^ lineage cells were present in the nasal membrane. Once the membrane was lifted, *Krt14*^*+*^*Ctsk*^*+*^ lineages proliferated considerably, continued to produce progeny cells and contributed to MSFL-induced osteogenesis. The dual recombinases-mediated lineage tracing based on Cre and Dre was first reported by He et al.^56^ By labeling different cell lineages with a combination of Cre-loxp and Dre-rox systems to activate respective fluorescent reporter genes, this technique is used to study the interactions between cells of different lineages.^57–65^ Although two different lineages could be identified using this technique, no labeling technique is available currently for exploring their respective heterogeneities simultaneously. Theoretically, the insertion of rox-stop-rox element at the 5′-end of the original loxp locus in Confetti mouse could enable us to investigate the heterogeneity of *Krt14*^*+*^*Ctsk*^*+*^ cells from the perspective of *Krt14* or *Ctsk*, respectively. This requires future works.

In addition, we depleted *Krt14*^*+*^ or *Ctsk*^*+*^ cells in *Krt14*-iDTR and *Ctsk*-iDTR mice, which further validated the results of the dual recombinases-mediated linage tracing experiment. Preoperative deletion of postnatally labeled *Krt14*^*+*^ cells (TAM at P1–P3) resulted in a complete loss of MSFL-induced bone regeneration, suggesting that during the neonatal period, *Krt14*^*+*^*Ctsk*^*+*^ cells might be the predominant osteoprogenitor cells in SM. In contrast, preoperative deletion of late-labeled *Krt14*^*+*^ cells (TAM at P6w) showed declined effect on the MSFL-induced bone regeneration, suggesting a possible differentiation from *Krt14*^*+*^*Ctsk*^*+*^ cells to *Krt14*^*–*^*Ctsk*^*+*^ cells during development. As indicated by scRNA-seq, Krt14 was expressed in both basal cells and the *Krt14*^*+*^*Ctsk*^*+*^ subset in OLCs, while Ctsk was expressed exclusively in all OLCs, including the *Krt14*^*+*^*Ctsk*^*+*^ cells. Given that the lineage of *Krt14*^*+*^ basal cells is not osteogenic, the *Krt14*-iDTR-based in vivo depletion confirmed the osteogenic potential of *Krt14*^*+*^*Ctsk*^*+*^ lineage cells and their contribution in MSFL-induced osteogenesis. Overall, the impacts of *Ctsk*^*+*^ cell deletion on osteoclasts and general effects of *Krt14*^*+*^ cell deletion were not specifically related to the lineage under investigation. However, their possible effects contributing to the model results could not be formally excluded. The *Rosa26*^*RSR-LSL-tdTomato-2A-iDTR*^ mouse line would be applied in the future to fulfil the study concerning the function of *Krt14*^*+*^*Ctsk*^*+*^ lineage cells from a more rigorous perspective.^66^

Apart from the transnasal MSFL model, we also observed *Krt14*^*+*^*Ctsk*^*+*^ cells in the MSFL model generated via the transalveolar approach, and these cells co-expressed epithelial (*Epcam*) and osteogenic (*Runx2*) markers (Supplemental information, Fig. S4), suggesting similar characteristics and functions of *Krt14*^*+*^*Ctsk*^*+*^ cells in maxillary sinus and the nasal cavity. Our results suggest that the regeneration of maxillofacial bones covered by mucosa (nasal bone and maxilla) is governed by a subset of cells with dual epithelial and mesenchymal properties and characteristics of osteoprogenitors. Our study, derived from clinical observations, enriches the theory of osteogenesis and provides more possibilities for clinical applications in guiding bone regeneration and tissue repair.

## Materials and methods

### Human specimens

The three healthy SM tissues used in this study were collected during orthognathic surgery at the Department of Oral and Maxillofacial Surgery of the Stomatological Hospital of Tongji University. In this surgery, local SMs were trimmed and removed; and the three specimens were from male donors aged 24, 25, and 27 years, respectively. All patients signed their informed consent in which all procedures of the study were detailed. The study was reviewed and approved by the Research Ethics Committee of Stomatological Hospital and Dental School of Tongji University (approval number: [2018]-SR-86).

### Study samples

From January 2010 to December 2020, 1210 patients (565 men and 645 women) aged between 18 and 75 years receiving MFSL and implant repair in the Department of Oral Implantology, Stomatological Hospital of Tongji University were enrolled. The inclusion criteria were: (a) no pathological changes in the sinus floor according to the preoperative CBCT scan; (b) MSFL and crown repair were performed; (c) the SM remained intact according to the postoperative CBCT scan. The exclusion criteria were patients with maxillary sinus cyst or sinusitis, or with metal or ceramic prostheses near the surgical area that may cause X-ray artifacts. All patients signed their informed consent in which all procedures of the study were detailed. The study was reviewed and approved by the Research Ethics Committee of Stomatological Hospital and Dental School of Tongji University (approval number: [2018]-SR-86).

### Image file preparation and measurements

All enrolled patients had CBCT scans (3D Accuitomo 170; J. Morita, Tokyo, Japan) performed at three time points: pre-surgery, immediately after the surgery, 6 months after the surgery. Technical parameters of all scans were: 87 kV, 5.5 Ma, exposure time 17.5 s. The residual bone height H0 was the distance from the initial sinus floor to the alveolar crest before surgery. H1 and H2 represent the distance from the highest point of the lifting SM to the alveolar crest immediately and 6 months after the operation, respectively. All measurements were performed three times and averaged. Osteogenic efficiency = (H2 – H0)/(H1 – H0).

### Animal models

*Igs2*^*em1(CAG-LSL-ZsGreen-wpre-pA-CAG-RSR-tdTomato-wpre-pA)Smoc*^ (*H11*^*LSL-ZsGreen-RSR-tdTomato*^) (NM-KI-200319), *Krt14*^*em2(CreERT2-Wpre-polyA)Smoc*^ (*Krt14*^*CreER*^) (NM-KI-190024), *Krt14*^*em3(2A-DreERT2)Smoc*^ (*Krt14*^*DreER*^) (NM-KI-190125), *Ctsk*^*em1(2A-CreERT2-WPRE-polyA)Smoc*^ (*Ctsk*^*CreER*^) (NM-KI-200067) with C57BL/6 background were purchased from the Shanghai Model Organisms Center. B6-*Gt(ROSA)26Sor*^*tm2(CAG-LSL-DTR-EGFP)*^ (*R26*^*CAG-LSL-iDTR-EGFP*^) (BCG-TO-0001) mice with C57BL/6 background were obtained from Biocytogen. B6.*Cg-Gt(ROSA)26Sor*^*tm14(CAG-tdTomato)Hze/J*^ (*R26*^*LSL-tdTomato*^) (007914) was acquired from Jackson Laboratory. *H11*^*LSL-ZsGreen-RSR-tdTomato*^*;Ctsk*^*CreER*^*;Krt14*^*DreER*^ mice were generated by crossing *Krt14*^*DreER*^ and *Ctsk*^*CreER*^ with *H11*^*LSL-ZsGreen-RSR-tdTomato*^ mice to excise the rox- and loxP-flanked stop cassette, respectively. In addition, *Krt14*^*CreER*^ or *Ctsk*^*CreER*^ mice were crossed with *R26*^*CAG-LSL-iDTR-EGFP*^ mice to generate iDTR mouse models. *Krt14*^*CreER*^ mice were crossed with *R26*^*CAG-LSL-tdTomato*^ mice to generate *Krt14*^*CreER*^*;R26*^*LSL-tdTomato*^ mice. All mouse procedures were approved by the Institutional Animal Care and Use Committee of Tongji University.

### MSFL

Mice were anaesthetized with 2% isoflurane. For the MSFL by transnasal approach, the nasal skin was shaved and scrubbed with 75% ethanol. A #11 blade was used to create a median incision. A 1/2 tungsten steel round bur was used to drill holes in the center of the nasal bones without breaking the nasal membrane. A mini implant (WEGO) with diameter of 0.6 mm and length of 0.8 mm was implanted with a mini screw. A 5 mm × 5 mm × 0.1 mm Teflon film was inserted between the bone surface and nasal tissue to maintain the stability of implants and avoid the infiltration of fibroblasts. The cut was sutured using 5–0 sutures. For the transalveolar approach, the maxillary first molar was extracted with the flap operation of the surrounding palatal mucosa. The same bur was used for drilling holes, while the mini implants with the length of 0.4 mm were applied, due to the anatomical size limitations of maxillary sinus. Mice were euthanized for analysis in accordance with the experimental design.

### Tissue preparations for LCM-based bulk RNA-seq

LCM-based bulk RNA-seq for hard tissue was a pipeline constructed for the acquisition of tissue transcriptome with spatial information with modifications of Geo-Seq.^67,68^ A specialized treatment pipeline was constructed to maintain the structure and RNA integrity of the hard tissues. The hard tissues were treated with a solution (2% formic acid, 5 M NaCl, 100 mM RVC, 50 mM sodium citrate, 20% deionized formamide, 20% ammonium sulfate, and 0.1 M 2-(*N*-morpholino) ethane sulfonic acid sodium salt in RNase-free water) at 4 °C for 48 h. The hard tissue was flushed with RNase-free water, dehydrated with 30% sucrose (w/v), and prepared for cryosections of 15 μm thickness. Target regions were acquired by laser capture microdissection with RNA extracted by Trizol reagent (Thermo Fisher Scientific, 15596018) and precipitation assisted by glycogen (Thermo Fisher Scientific, R0561, 20 μg/mL).

### scRNA-seq

scRNA-seq libraries were prepared using the Chromium Next GEM Single Cell 3′ Kit version 3.1 (10× Genomics, 1000075), following the manufacturer’s instructions. In brief, single live cells were collected after the dissection and digestion processes as described in the “Flow cytometry of membranes and newly formed tissues in MSFL” section and resuspended in PBS containing 0.04% BSA (Sigma-Aldrich, A1933) to a final concentration of 500–1200 cells/mL as determined by a TC20 cell counter (BioRad). Ten thousand cells were captured in droplets to generate nanoliter-scale gel beads in EMulsion (GEMs). GEMs were then reverse transcribed in a C1000 Touch Thermal Cycler (Bio-Rad) programmed at 53 °C for 45 min, 85 °C for 5 min, and held at 4 °C. After reverse transcription and cell barcoding, emulsions were broken and cDNA was isolated and purified with Cleanup Mix containing DynaBeads (Thermo Fisher Scientific, 37002D) and SPRIselect reagent (Beckman, B23318), followed by PCR amplification. Amplified cDNA was then used for 3′ gene expression library construction, and single-cell RNA libraries were sequenced using an Illumina NovaSeq 6000 sequencer with 150 bp paired-end reads.

### scRNA-seq data processing

The Cell Ranger toolkit (version 3.1, 10× Genomics) was applied to aggregate raw data, filter low-quality reads, align reads to the mouse reference genome (mm10), assign cell barcodes, and generate a unique molecular identifier (UMI) matrix. Seurat (version 4.0.2) (https://satijalab.org/seurat/) was used to analyze scRNA-seq data. Specifically, the raw UMI matrix was processed to filter out genes detected in < 10 cells and cells with < 200 genes. We further quantified the number of genes and UMI counts for each cell and maintained high-quality cells with thresholds of 500–120,000 UMIs, 400–8000 genes. To screen out cells with high mitochondrial proportion, we used Seurat’s default parameter of 20% to ensure that most of the heterogeneous cell types were included for downstream analysis. In order to reduce the effect of mitochondrial genes on subsequent analysis, a regression was carried out before clustering in our study. Scrublet^69^ (https://github.com/AllonKleinLab/scrublet) was then applied to remove potential doublets with the expected doublet rate of 6%, and cells with doublet scores larger than 90% quantile were filtered out. The normalized expression matrix was calculated based on the raw UMI counts after normalizing the total counts per cell (library size) and then scaled by 1e6 and logarithmically transformed.

### Dimension reduction and unsupervised clustering for scRNA-seq data

Dimension reduction and unsupervised clustering were performed according to the standard workflow in Seurat. In brief, the dispersion-based methods were carried out to detect the top 20 highly variable genes (HVGs),^70^ where the normalized dispersion was obtained by scaling with the mean and standard deviation of the dispersions for genes falling into a given bin. The unwanted sources of variation, including total counts, percentages of mitochondrial gene counts and cell cycle-related gene counts were regressed out from the normalized expression matrices. For the clustering of 63,527 cells (> 60,000 cells) in total, PCA was performed on the variable gene matrix to reduce noise. Fifty components were calculated and the top 40 principal components were used for downstream analyses. Then, Leiden algorithm was applied to find cell clusters, with the parameter of resolution equaling 1,^71^ which referred to the construction of the database TISCH.^72^ Of note, the same principal components were also used for non-linear dimension reduction to generate the UMAP for visualization.

After the first-round unsupervised clustering, we annotated each cell cluster according to marker genes, and identified the major cell types including chondrocytes, OLCs, pericytes, neural progenitor cells, neutrophil, macrophage, epithelial cells, T cells, sustantacular cells, HBCs, iOSNs and mOSNs. Notably, OLC cluster derived from newly formed tissues would be separated clearly, and thus, we performed a second-round unsupervised clustering on the major cluster to obtain the high-resolution map of OLCs. The second-round clustering procedure was the same as the first-round clustering, both of which started from unfiltered expression matrix, and then identified HVGs, calculated PCA matrix, corrected batch effects by CCA2, detected cell clusters by Leiden algorithm and performed dimensionality reduction for visualization. All analyses were performed with Seurat.

To detect DEGs in specific clusters in OLCs, we normalized the raw count matrix by CCA2, which models the UMI counts using a regularized negative binomial model to remove the variation of sequencing depth, while adjusting the variance based on pooling information across genes with similar abundances. The DEGs were then detected by Limma package^73^ (https://bioinf.wehi.edu.au/limma/) with the normalized expression obtained via SCTransform (https://satijalab.org/seurat/archive/v3.0/sctransform_vignette.html).

### Bulk RNA-seq

For LCM-based bulk RNA-seq, tissues of MSFL models were treated as described in the “Tissue preparations for LCM-based bulk RNA-seq” section. The FACS-based acquirements of *Krt14*^+^*Ctsk*^+^, *Krt14*^+^*Ctsk*^–^ or *Krt14*^–^*Ctsk*^+^ cells from the adult mice, fetal mice or the SMs of human beings were described in the “FACS-based *Krt14*^+^*Ctsk*^–^, *Krt14*^+^*Ctsk*^+^ and *Krt14*^–^*Ctsk*^+^ cell sorting” section, with the RNA-seq pipeline carried out instantly without extra in vitro cell culture.

Followed by total RNA extraction, the RNA quality was determined using an Agilent 2100 Bioanalyzer, all groups had RNA integrity (RIN) numbers > 7.0, and the library construction was conducted according to the instructions of the low input Trio RNA-Seq^TM^ library preparation Kit (Nugen, 0357-32), with the insert length varying around 350 bp. Novaseq 6000 was used for high-throughput sequencing with the strategy of PE150. Raw reads were trimmed and aligned using Hisat (https://www.psc.edu/resources/software/hisat-2) and Samtools (http://www.htslib.org/doc/samtools.html) with the mm10 genome and normalized using TPM.^74^ The R package WGCNA was used to cluster the modules and analyze the core genes in the modules.^75^ Mfuzz (http://mfuzz.sysbiolab.eu) was used for time course analysis. A two-way hierarchical clustering heatmap using Euclidean distance and average linkage was used to display modules from the five groups. Cytoscape 3.7.1 (https://cytoscape.org/index.html) was used to depict the protein–protein interaction network. PCA was applied to display the correlations among different cell types. Spearman’s correlation was utilized to compare the similarities of transcriptomic features between two groups.

### TAM treatment

For induction of CreER in pregnancy, pregnant mice were injected with 750 µg TAM (Sigma, H6278, 10 mg/mL). For neonatal induction of CreER or DreER activity, mice were administered short-term oral dosing with TAM (50 mg/kg) on days 1–3 after birth. For induction of recombinase activity in juvenile mice, mice were intraperitoneally injected with 100 µL TAM (10 mg/mL) for 3 days at 6-week old or 7-week old in accordance with the experimental design.

### DT treatment

Due to the wide distribution of target cells, the mice died after a 2-day intraperitoneal administration of DT (Sigma, D0564). Continuous localized injection to the nasal membrane for 10 days at a dose of 3 μL (1 μg/μL) was used to induce *Krt14*^*+*^*/Ctsk*^*+*^ cell toxicity.

### Flow cytometry of membranes and newly formed tissues in MSFL

Nasal membranes of 40 mice, along with the newly formed tissues of 80 mice by MSFL on days 6 and 14 post-operation were dissected under a stereomicroscope. Tissues were incubated with digestion buffer (1 mL DPBS buffer containing 3 mg collagenase I, 3 mg dispase II, and 0.1 mg DNase I) in a shaking bath at 37 °C for 30 min. Digested cells were filtered with a 70 mm cell strainer (BD Falcon, 352350) to remove the remaining cell mass and centrifuged at 4 °C at 1500 rpm for 5 min. The supernatant was discarded, and cells were suspended in 500 μL red blood cell lysis buffer (eBioscience, 00433357) at 4 °C for 5 min. Equal amounts of staining buffer (DPBS containing 0.1% BSA) were used to neutralize the lysis buffer and centrifuged at 4 °C at 1500 rpm for 5 min. After resuspension with staining buffer, dead cells were removed using the dead cell removal kit (Miltenyi Biotec, 130090101) and live cells were fixed in 4% paraformaldehyde (PFA) for 15 min. Fixed cells were treated with 0.1% Triton X-100 (Sigma-Aldrich) for 1 min and washed twice with staining buffer. The fixed live permeabilized cells were suspended in 100 μL staining buffer and incubated for 30 min on ice with the following antibodies: mouse IgG2a-AF647 (Santa Cruz, sc-24637), mouse IgG3-PE (Santa Cruz, sc-2869), Krt14-AF647 (Santa Cruz, sc-53253) and Ctsk-PE (Santa Cruz, sc-48353), with the dilution of 1:400. Blank control, isotype control and samples labeled by single antibody were applied in the study. The validity of this intracellular staining workflow has been tested before formal experiments by cell lines, MC3T3-E1 and HEK293, which are positive for Ctsk and Krt14, respectively.

### FACS-based *Krt14*^*+*^*Ctsk*^*–*^, *Krt14*^*+*^*Ctsk*^*+*^ and *Krt14*^*–*^*Ctsk*^*+*^ cell sorting

Live cells were collected by FACS-based sorting for in vitro culture, viability test, osteogenic induction and RNA-seq. To acquire live mouse *Krt14*^*+*^*Ctsk*^*–*^, *Krt14*^*+*^*Ctsk*^*+*^ and *Krt14*^*–*^*Ctsk*^*+*^ cells, the local tissues of 20 adult dual reporter mice (TAM on MSFL days 2–4 and sampling on day 6, 50 mg/kg) were dissected and digested in accordance with the steps described in the “Flow cytometry of membranes and newly formed tissues in MSFL” section without fixation, permeablization and antibody labeling. To identify whether similar cell types exist in the embryonic period, the local tissues of fetal mice were treated in the same way as that described above through the administration of TAM at E14.5 (750 μg/pregnant mouse) and sampling at E15.5.

For collecting similar cell types in SMs of human being, the dissected specimen was first transfected by lentivirus, which carried the promoters of human genes of *KRT14* (NM_000526.5) and *CTSK* (NM_000396.4), followed by reporter elements of tdTomato and EGFP, respectively. Similar treatment pipeline was carried out 72 h after transfection as described in the previous part and the labeled cells were sorted based on the fluorescent reporter signals.

### Multicolor immunohistochemistry and confocal imaging

The process of tissue treatment was described in our prior study.^76^ In brief, tissues were fixed in 4% PFA for 24 h and decalcified with 10% EDTA solution for 1 month at 4 °C. Paraffin blocks were cut into 4 mm sections and adhered to a glass slide heated at 70 °C for 1 h; deparaffinized in xylene; and then rehydrated in 100%, 95%, 85%, 70%, and 50% alcohol in sequence. Multiplex immunofluorescence staining was performed using TSA-based system (Thermo Fisher Scientific). In brief, antigens were retrieved using citric acid solution (pH 9.0) and boiled in an oven for 15 min. After pre-incubation with blocking buffer at room temperature for 15 min, multicolor immunohistochemistry was performed with sequential incubation of different primary antibodies, secondary antibodies, and TSA reagents with different fluoresceins. With the accumulation of fluorescent signals, the previous round of antibodies were stripped, followed by the incubation of the next round of antibodies. Nuclei were stained with DAPI after all the antigens were labeled. The antibodies used in the immunofluorescence or multicolor immunohistochemistry included rabbit anti-mouse/human Krt14 (Novus Biologicals, NBP2-67585, 1:400), goat anti-mouse/human Vim (R&D systems, AF2105, 1:400), rabbit anti-mouse/human Ctsk (Abcam, ab19027, 1:200), rabbit anti-mouse Runx2 (Cell Signaling Technology, 12556, 1:200), mouse anti-mouse/human E-cadherin (Abcam, ab231303, 1:200), rabbit anti-mouse/human Alpl (Thermo Fisher, MA5-24845, 1:200), rabbit anti-mouse/human Bglap (Abcam, ab93876, 1:200). Images were acquired using a Nikon TI2-E^+^A1 R microscope with the adjustable wavelength receiving module to separate the spectra of the channels and avoid overlap.

### Cell viability assay

Cell proliferation and viability was measured with cell counting kit-8 (CCK-8) assay (Vazyme, A311-01) in accordance with the instructions. Briefly, cells of each groups (2000/well) were seeded in 96-well plates, with the time points for analyses on days 1, 3, 5, and 7. The absorbance at 450 nm was measured by a hybrid multi-mode microplate reader (Synergy H1, BioTek, USA).

### In vitro osteogenic differentiation

FACS-sorted cells were cultivated in osteogenic differentiation-inducing medium containing 10% FBS αMEM supplemented with 100 IU/mL penicillin-streptomycin, 50 µg/mL ascorbic acid, 10 nM dexamethasone and 5 mM β-glycerol phosphate. After 4-week induction, cells were fixed with 4% PFA for 15 min, stained with 40 mM Alizarin red solution (Millipore, 2003999) for 15 min, and rinsed five times.

### Quantification and statistical analysis

Independent two-tailed Student’s *t*-tests, one-way ANOVA or two-way ANOVA were used to analyze data after determining whether data were normally distributed. Experiments were performed using at least three independent samples except for the flow cytometry-based cell identification. Differences were considered statistically significant at *P* values < 0.05. Unless specifically stated, data are represented as means ± SD. The statistical analysis was conducted using IBM SPSS Statistics version 26.0 (IBM Corp., Armonk, NY, USA).

## Supplementary information

Supplementary information, Fig. S1

Supplementary information, Fig. S2

Supplementary information, Fig. S3

Supplementary information, Fig. S4

Supplementary information, Fig. S5

Supplementary information, Fig. S6

Supplementary information, Table S1

## Data Availability

All of the scRNA-seq and bulk RNA-seq data have been deposited in the NGDC database, with accession numbers: HRA002548, CRA007231.
