# Supplementary material for: A novel lineage of osteoprogenitor cells with dual epithelial and mesenchymal properties govern maxillofacial bone homeostasis and regeneration after MSFL
Source: Cell Res. 2022 Jul 12;32(9):814–30. doi: 10.1038/s41422-022-00687-x (PMC9436969; doi:10.1038/s41422-022-00687-x)
Supplement: Supplementary file 1 — Supplementary information, Fig. S1 [file 41422_2022_687_MOESM1_ESM.pdf]

Figure S1

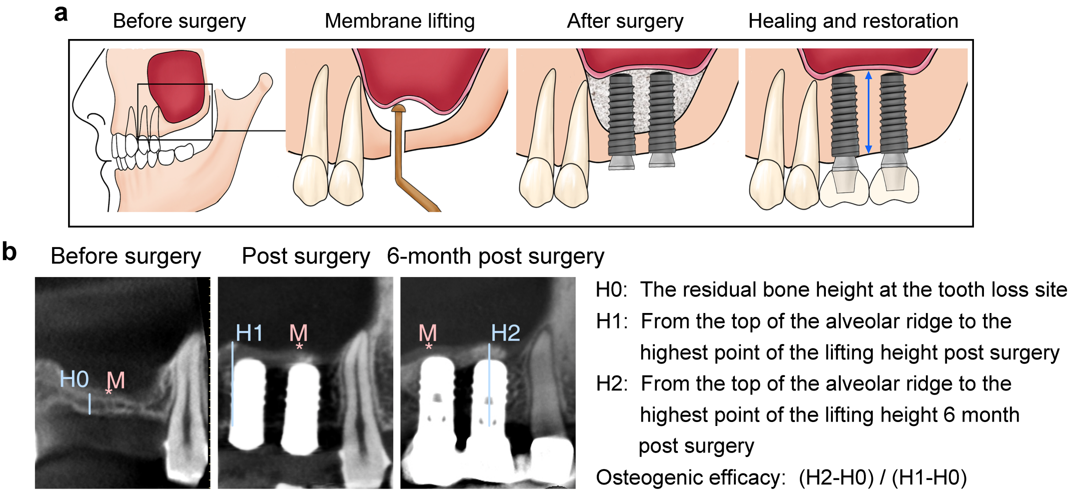

c

Clinical evaluation of MSFL osteogenic efficacy (n=1210)

	n (%) or mean ± SD	Osteogenic efficacy (mean ± SD)	*p* value
**Gender**			0.64
Male	565 (46.69%)	0.87 ± 0.25	
Female	645 (53.31%)	0.86 ± 0.20	
**Age (years)**			0.80
<30	25.55 ± 3.25	0.88 ± 0.31	
30-39	35.29 ± 2.49	0.86 ± 0.19	
40-49	44.65 ± 2.97	0.87 ± 0.23	
50-59	54.01 ± 2.77	0.86 ± 0.24	
>60	64.64 ± 3.67	0.86 ± 0.15	

SD, standard deviation;

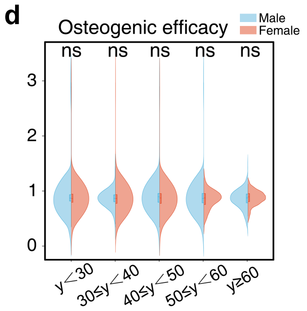

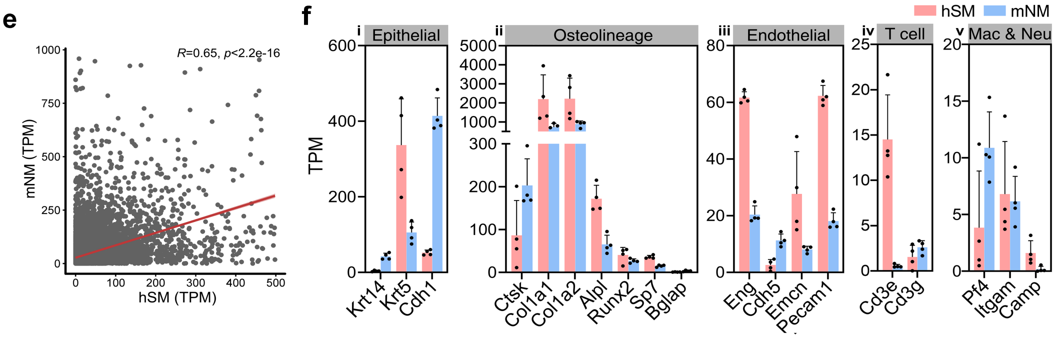

Supplementary information Fig. S1 Clinical procedure of MSFL and analysis of 1,210 clinical cases.

a Schematic diagram showing the surgical process of MSFL.

b Representative CBCT images of before surgery (left), immediately after surgery (middle), and 6 month after surgery (right); M: membrane; H0: the residual bone height at the tooth loss site; H1: from the top of the alveolar ridge to the highest point of the lifting height immediately post surgery; H2: from the top of the alveolar ridge to the highest point of the lifting height 6 month post surgery; Osteogenic efficacy: (H2-H0) / (H1-H0).

c Analysis of MSFL osteogenic efficacy in different age group and gender (n=1,210). Data were compared with two-way ANOVA.

d Violin plots generated from **a,** showing osteogenic efficacy separated by age and gender; ns: no significance.

e Correlation analysis of hSM and mNM at transcriptomic level. TPM of genes were analyzed by Spearman correlation analysis. R=0.65, p<2.2e-16.

f Comparison of TPM of T cell, macrophage, neutrophil, epithelial cell, osteolineage cell, and endothelial cell specific markers in hSM and mNM. error bars indicated SEM.
