# Supplementary material for: A novel lineage of osteoprogenitor cells with dual epithelial and mesenchymal properties govern maxillofacial bone homeostasis and regeneration after MSFL
Source: Cell Res. 2022 Jul 12;32(9):814–30. doi: 10.1038/s41422-022-00687-x (PMC9436969; doi:10.1038/s41422-022-00687-x)
Supplement: Supplementary file 2 — Supplementary information, Fig. S2 [file 41422_2022_687_MOESM2_ESM.pdf]

Figure S2

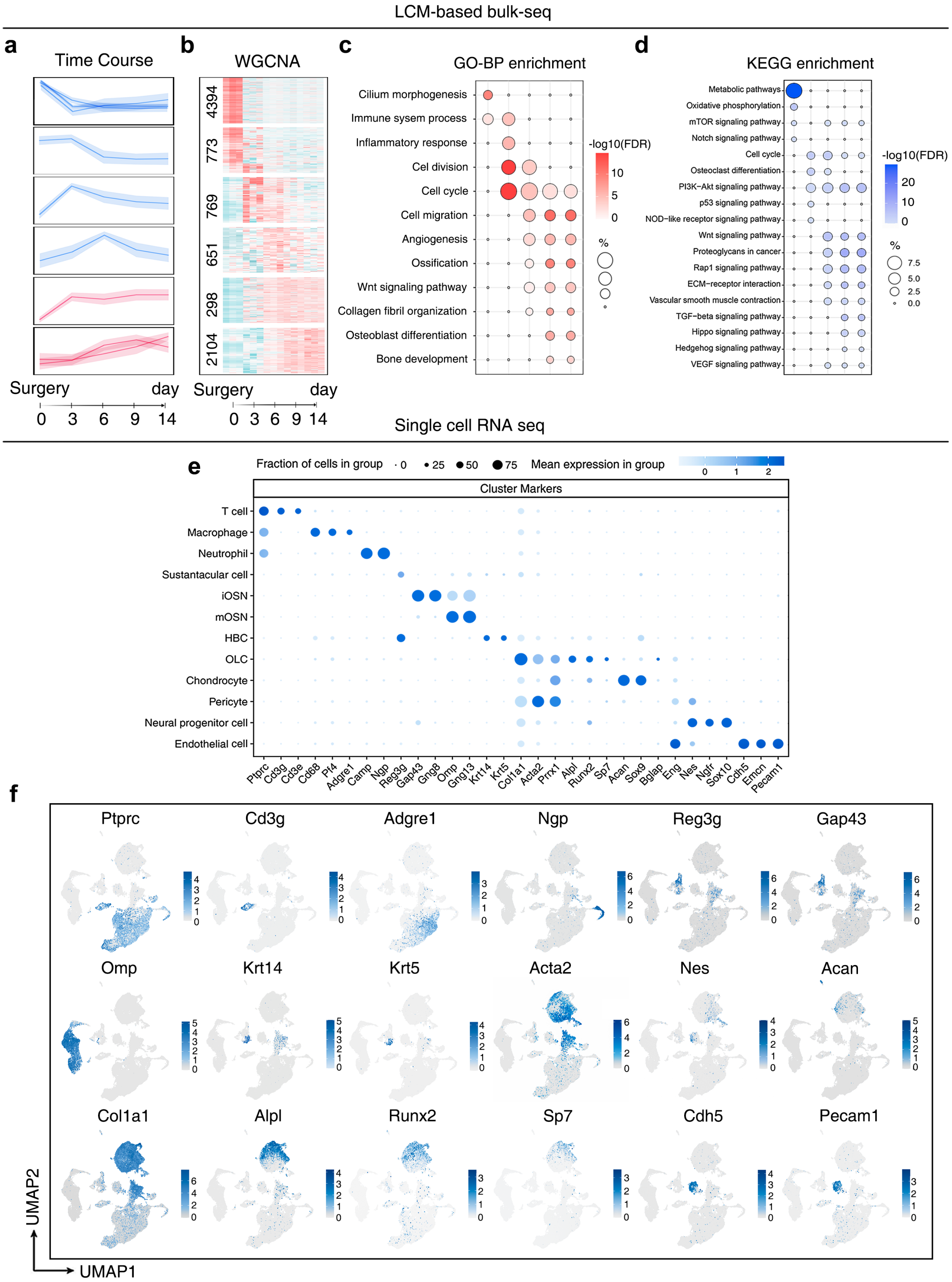

Supplementary information Fig. S2 LCM-based bulk-seq and scRNA-seq.

a Time course analysis.

b Heatmap of modules analyzed using WGCNA.

GO-BP (**c**) and KEGG enrichment (**d**) of genes from representative modules at each time point.

e–f Expression patterns of cluster signature genes.
