# Supplementary material for: A novel lineage of osteoprogenitor cells with dual epithelial and mesenchymal properties govern maxillofacial bone homeostasis and regeneration after MSFL
Source: Cell Res. 2022 Jul 12;32(9):814–30. doi: 10.1038/s41422-022-00687-x (PMC9436969; doi:10.1038/s41422-022-00687-x)
Supplement: Supplementary file 3 — Supplementary information, Fig. S3 [file 41422_2022_687_MOESM3_ESM.pdf]

Figure S3

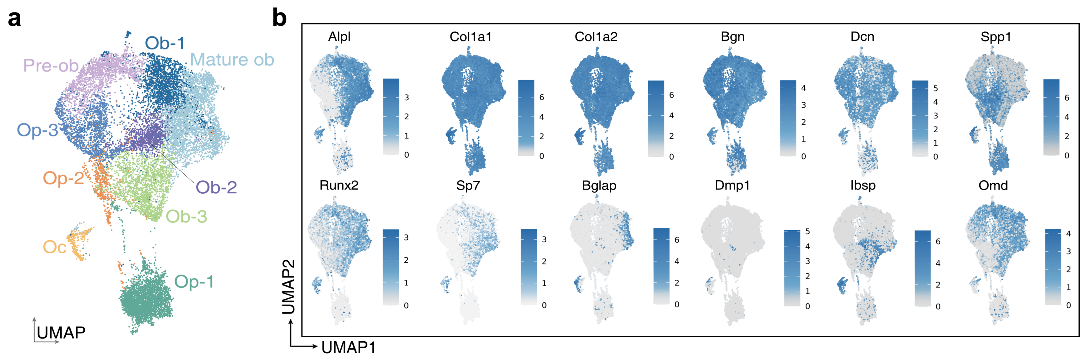

Supplementary information Fig. S3 OLCs subclustering based on scRNA-seq.

a Reclustering of all OLCs, shown in UMAP embedding and color coded by subclusters.

b Distribution of osteogenic-related genes visualized on UMAP projection. color coded by the expression level of each gene.
