# Supplementary material for: A novel lineage of osteoprogenitor cells with dual epithelial and mesenchymal properties govern maxillofacial bone homeostasis and regeneration after MSFL
Source: Cell Res. 2022 Jul 12;32(9):814–30. doi: 10.1038/s41422-022-00687-x (PMC9436969; doi:10.1038/s41422-022-00687-x)
Supplement: Supplementary file 4 — Supplementary information, Fig. S4 [file 41422_2022_687_MOESM4_ESM.pdf]

Figure S4

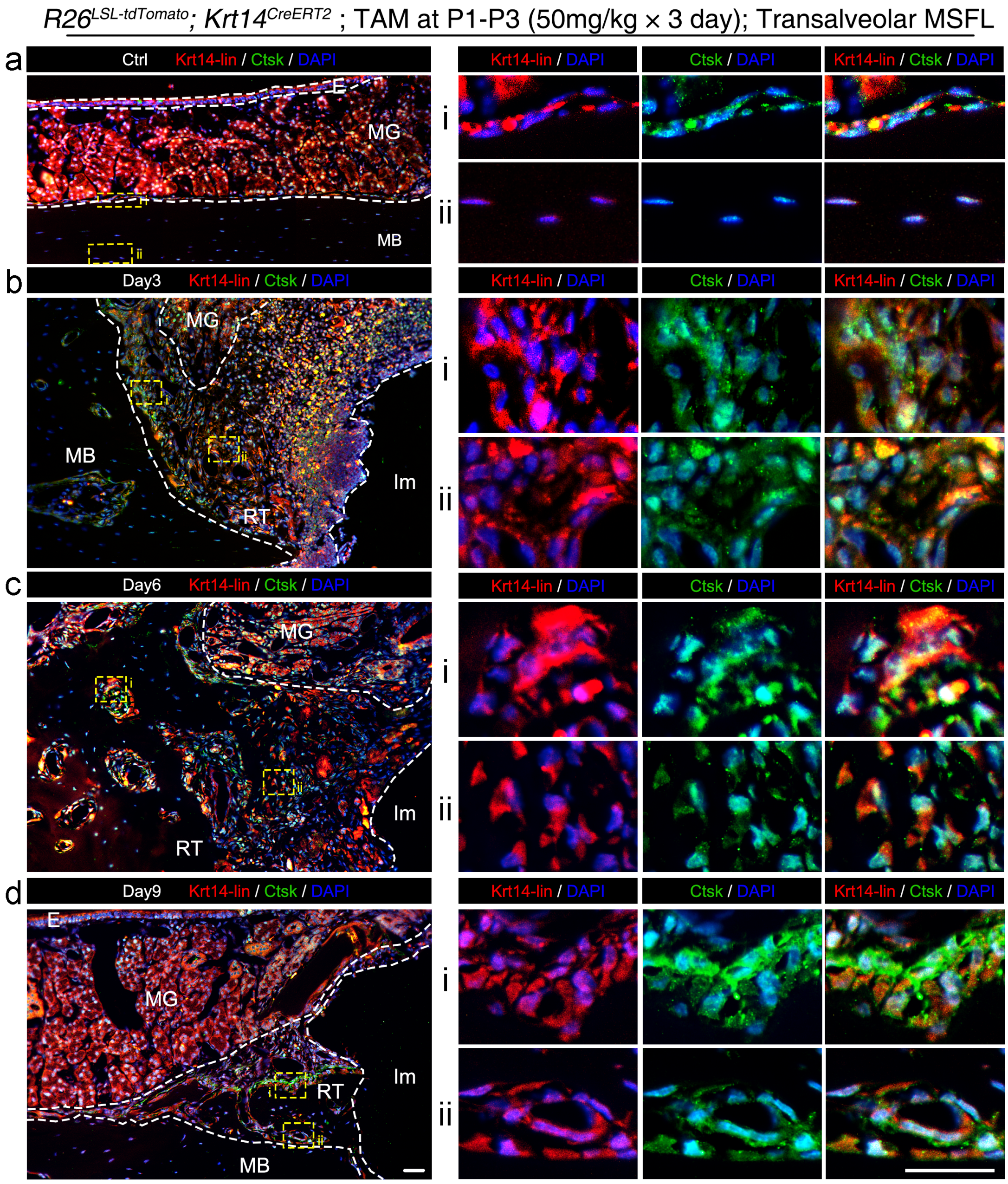

Supplementary information Fig. S4 ***Krt14⁺Ctsk⁺*** **cells contributed to maxillary bone turnover and transalveolar MSFL induced osteogenesis.**

Representative confocal images of sample sections from Ctrl(a), days 3(b), day 6(c), day 9(d) of transalveolar MSFL models. Area in the box is magnified in the right of each panel. Merged and single-channel images of Krt14 lineage (red), Ctsk (green), and DAPI (blue) were shown. White dotted line indicating the boundaries of anatomical markers. E, epithelium. MG, maxillary gland. MB, maxillary bone. RT, regenerative tissue. Im, the possition of mini-implants. Bar=50um; lin: lineage; n = 3 from 3 independent experiments.
