# Supplementary material for: A novel lineage of osteoprogenitor cells with dual epithelial and mesenchymal properties govern maxillofacial bone homeostasis and regeneration after MSFL
Source: Cell Res. 2022 Jul 12;32(9):814–30. doi: 10.1038/s41422-022-00687-x (PMC9436969; doi:10.1038/s41422-022-00687-x)
Supplement: Supplementary file 5 — Supplementary information, Fig. S5 [file 41422_2022_687_MOESM5_ESM.pdf]

Figure S5

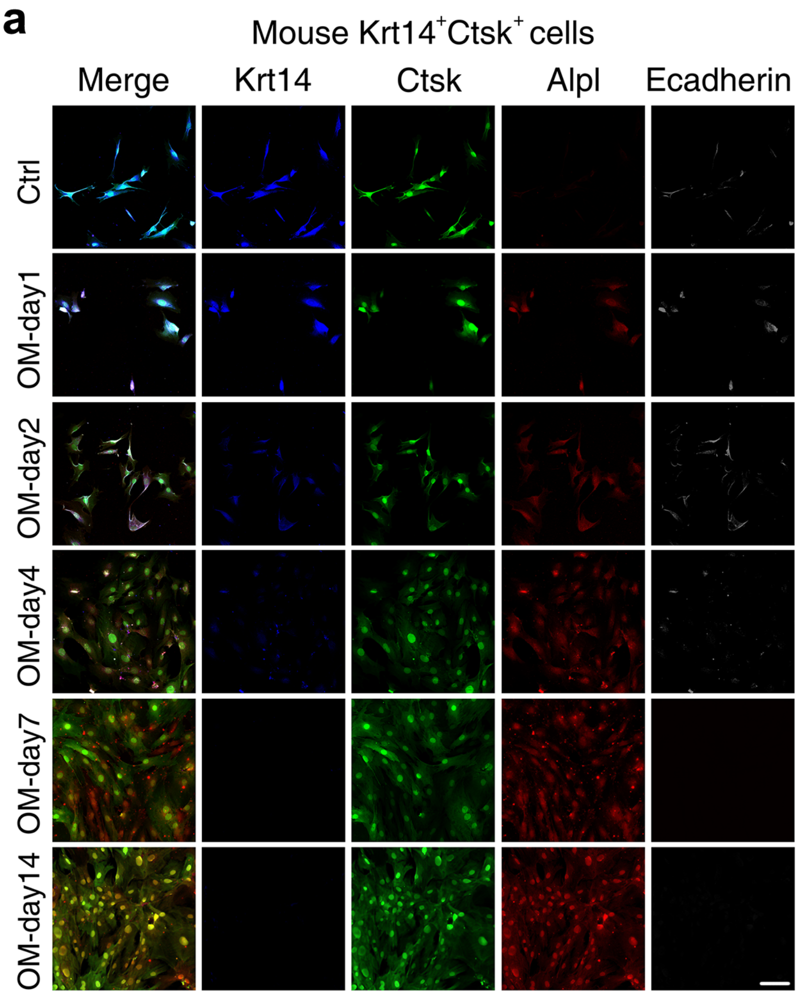

Supplementary information Fig. S5 Osteogenic differentiation ability of mouse *Krt14*$^{+}$ *Ctsk*$^{+}$ cells *in vitro.*

Representative confocal images of mouse *Krt14*$^{+}$*Ctsk*$^{+}$ cells cultured in OM on Ctrl, days 1, 2, 4, 7, and 14. Merged and single-channel images of Ctsk (green), Krt14 (blue), Alpl (red), and Ecaderin (grey) are shown in each panel; bar=25 μm. n = 3 from 3 independent experiments.
