# Supplementary material for: A novel lineage of osteoprogenitor cells with dual epithelial and mesenchymal properties govern maxillofacial bone homeostasis and regeneration after MSFL
Source: Cell Res. 2022 Jul 12;32(9):814–30. doi: 10.1038/s41422-022-00687-x (PMC9436969; doi:10.1038/s41422-022-00687-x)
Supplement: Supplementary file 6 — Supplementary information, Fig. S6 [file 41422_2022_687_MOESM6_ESM.pdf]

SFigure 6

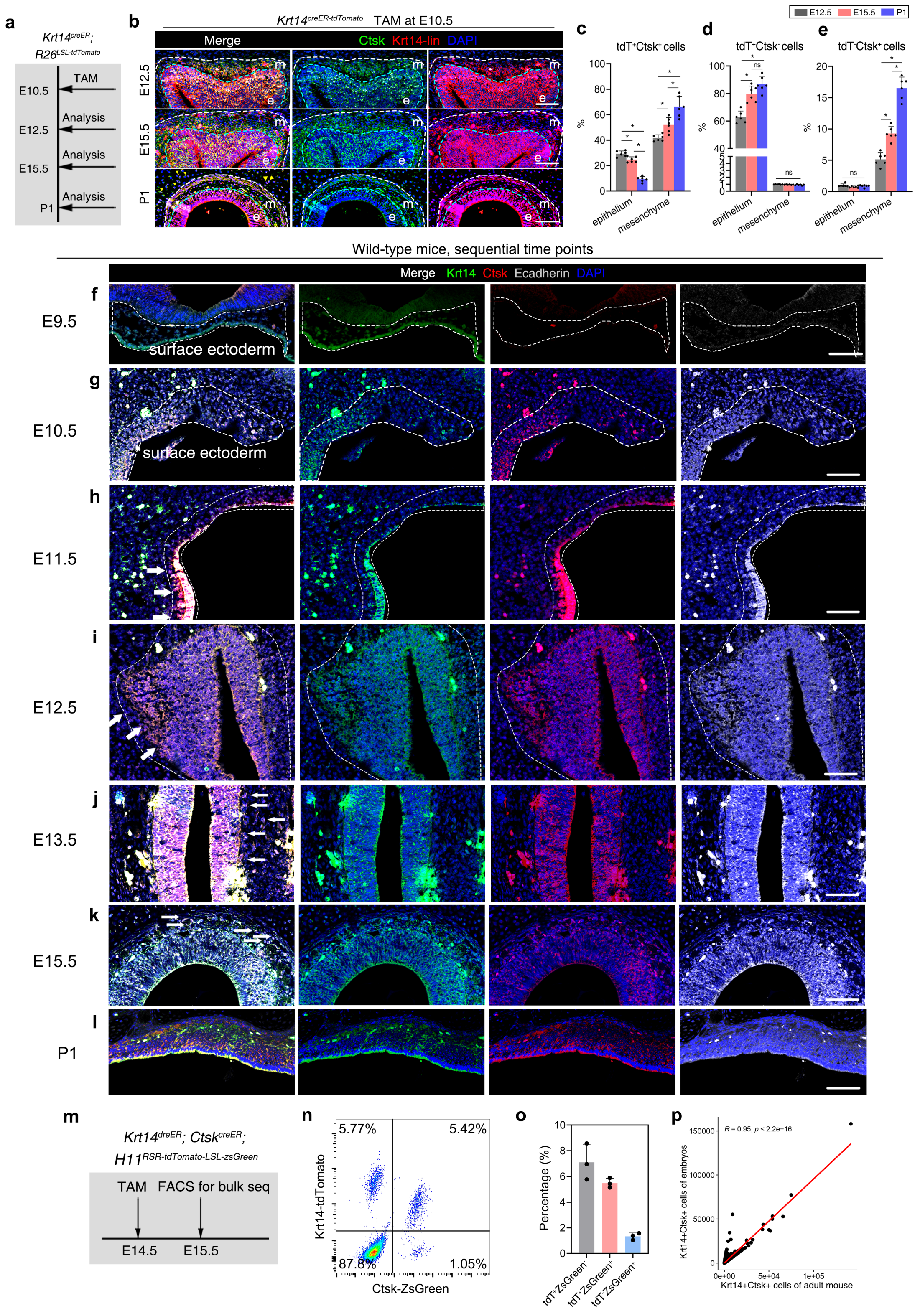

Supplementary information SFig. 6 *Krt14*$^{+}$*Ctsk*$^{+}$ cells Firstly Appeared at E12.5.

a TAM treatment strategy with *Krt14*^{CreER}*; R26*$^{LSL-\ tdTomato}$ mice to perform lineage tracing.

b Representative confocal images of local nose sections from *Krt14*^{creER}*; R26*$^{LSL-tdTomato}$ mice at E12.5, E15.5, and P1, with tamoxifen (TAM) treatment at E10.5. Merged and single

channel images of Krt14-lin (red), Ctsk (green), and DAPI (blue) are presented in each panel. n = 3 mice per condition from 3 independent experiments. The white dotted line depicts the boundary of the future lower layer of the Schneiderian membrane and the nasal bone. The cyan dotted line depicts the boundary of the epithelium and the lower layer of the future Schneiderian membrane. Yellow triangles indicating the tdT^{+}*Ctsk*$^{+}$ cells in the future maxillary bone. m, mesenchyme; e, epithelium; bar=50 μm.

c–e Statistical analysis of tdT^{+}*Ctsk*$^{+}$, tdT^{+}*Ctsk*$^{-}$, and tdT^{-}*Ctsk*$^{+}$ cells in the local regions. Data are presented as the mean ± SD of 5 mice per treatment from 3 independent experiments. One-way ANOVA was used to compare the percentage of the cells, followed by Dunnett's multiple comparisons test; ns: no significance; *$p < 0.05$.

f–l Representative confocal images of local nose sections from normal E9.5, E10.5, E11.5, E12.5, E13.5, and E15.5 in wildtype mice. Merged and single channel images of Krt14 (green), Ctsk (red), Ecadherin (grey), and DAPI (blue) are shown in each column. n = 3 mice per condition from 3 independent experiments. Bar = 50 μm. Dash lines (**f-l**) indicating the regions of the surface ectoderm. Arrows (**h-k**) indicating the *Krt14*$^{+}$*Ctsk*$^{+}$ cells.

m TAM strategy with *Krt14*^{DreER}*; Ctsk*^{CreER}*; H11* $^{RSR\text{-}tdTomato\text{-}LSL\text{-}ZsGreen}$ mice for FACS sorting.

n FACS sorted tdT^{+}zsGreen^{-} cells, tdT^{+}zsGreen^{+}cells, and tdT^{-}zsGreen^{+}cells; n = 5 mice per condition from 3 independent experiment.

o Histogram generated from **n;** mean ± SD.

p Correlation analysis of human and mouse *Krt14*$^{+}$*Ctsk*$^{+}$ at transcriptomic level. TPM of genes were analyzed by Spearman correlation analysis. R=0.95, p<2.2e-16.
