# Supplementary material for: A novel lineage of osteoprogenitor cells with dual epithelial and mesenchymal properties govern maxillofacial bone homeostasis and regeneration after MSFL
Source: Cell Res. 2022 Jul 12;32(9):814–30. doi: 10.1038/s41422-022-00687-x (PMC9436969; doi:10.1038/s41422-022-00687-x)
Supplement: Supplementary file 7 — Supplementary information, Table S1 [file 41422_2022_687_MOESM7_ESM.pdf]

Supplementary Table 1. Differential expressed genes included in GSEA analysis (Krt14$^+$ cells in Op-1 vs Krt14$^+$ cells in HBC), Related to Figure 3.

GSEA analysis

Description	Genes included
**Epithelial to mesenchymal transition**	Col1a1 S100a4 Sfrp2 Snai2
**Ossification**	Col1a1 Col1a2 Spp1 Ctsk Mmp13 Sparc Col6a1 Mmp2 Mgp Sfrp2 Cthrc1 Aspn Cdh11
**Extracellular matrix organization**	Col1a1 Col1a2 Spp1 Col3a1 Bgn Lum Ctsk Mmp13 Sparc Col6a1 Mmp2 Postn Eln Col6a3 Col5a1 Ctss Serpinh1 Sfrp2 Col6a2 Mfap2 Cst3 Fn1 Mfap4 Col8a2 Mfap5
**Skeletal system development**	Col1a1 Col1a2 Col3a1 Bgn Lum Ctsk Mmp13 Sparc Tyrobp Mmp2 Mgp Serpinh1 Sfrp2 Cdh11 Pdgfrb Prrx1
**Multicellular organism development**	Col1a1 Col1a2 Sparc Vim Col3a1 Bgn Lum Ctsk Acta2 Lgals1 Mmp13 Sparc S100a9 Tyrobp Col6a1 Igfbp7 Tagln Mmp2 Selenom S100a4 S100a8 Postn Eln Serpinf1 Mgp Crip1 Apoe Col6a3 Col5a1 Serpinh1 Selenop Aqp1 Sfrp2 Rcn3 Cthrc1 Mxra8 Itm2a Aspn Mfap2 Cst3 Cdh11 Pdgfrb Trem2 Prrx1 Fn1 Myl6 Ptgis
**Epidermal cell differentiation**	Slc4a7 Hes1 Krt15 Krt7 Krt6a Krt19 Sprr1a Krt8 Krt18 Dsp Perp Krt17 Krt5 Anxa1 Aqp3 Sfn
**Keratinization**	Krt15 Krt7 Krt6a Krt19 Sprr1a Krt8 Krt18 Dsp Perp Krt17 Krt5 Sfn

Information for included genes

Gene Name	p_val	avg_log2FC	pct.1	pct.2
Col1a1	5.99E-148	4.872045603	0.992	0.482
Dsp	1.99E-145	-2.014091903	0.011	0.908
Perp	1.61E-136	-2.136744422	0.051	0.967
Aqp3	6.25E-131	-2.798406944	0.04	0.897
Sfn	3.85E-125	-2.663199235	0.126	0.978
Lgals1	4.29E-121	2.599355898	0.983	0.746
Krt5	1.94E-116	-2.763968638	0.093	0.895
Col1a2	4.16E-113	3.655300899	0.924	0.487
Sparc	7.10E-108	2.661327342	0.941	0.788
Slc4a7	2.46E-107	-1.227929119	0.019	0.763
Spp1	6.60E-104	3.536223902	0.909	0.625
Krt17	9.49E-102	-2.729707947	0.274	0.98
Col3a1	2.79E-101	3.635924639	0.867	0.373

Vim	5.10E-101	3.404646622	0.88	0.469
Apoe	2.25E-98	2.314384914	0.966	0.855
Krt7	1.35E-97	-1.732272466	0.074	0.842
Mgp	7.22E-94	2.008104925	0.979	0.676
Krt18	5.23E-90	-2.161404908	0.196	0.975
Krt8	5.97E-86	-1.820356384	0.164	0.949
Krt19	1.03E-79	-1.646942146	0.093	0.781
Acta2	2.16E-75	2.872412298	0.785	0.335
Hes1	1.42E-72	-1.114914039	0.133	0.85
Lum	1.21E-63	3.114375467	0.657	0.196
Snai2	2.18E-62	-0.520731968	0.021	0.525
Bgn	1.99E-58	3.093164367	0.642	0.219
Anxa1	1.90E-56	-2.722773164	0.343	0.942
Krt6a	3.52E-51	-2.062023354	0.027	0.458
Myl6	2.35E-49	1.235097671	0.891	0.989
Sprr1a	4.30E-46	-2.039523452	0.017	0.402
S100a9	6.94E-34	3.540871936	0.585	0.353
Ctsk	1.39E-32	2.585392556	0.408	0.103
Krt15	1.32E-28	-1.154529895	0.004	0.243
Cst3	8.81E-26	1.717264199	0.701	0.938
Tyrobp	2.12E-24	2.671270275	0.484	0.27
Tagln	4.54E-24	2.159072552	0.526	0.324
Mmp13	1.57E-23	2.706388266	0.398	0.156
Fn1	9.70E-21	1.294879337	0.691	0.685
Selenom	1.08E-19	2.383705951	0.425	0.234
S100a4	1.43E-19	2.47564531	0.472	0.312
Igfbp7	3.08E-16	2.264216181	0.512	0.44
Col6a1	5.04E-16	2.1336715	0.352	0.17
Mmp2	5.82E-16	2.315420622	0.375	0.203
S100a8	1.33E-15	3.297334736	0.453	0.326
Crip1	3.02E-15	2.102803975	0.602	0.734
Col5a1	6.33E-15	1.900988923	0.269	0.094
Col6a3	1.83E-12	2.049956474	0.282	0.127
Postn	4.58E-12	2.005941945	0.276	0.125
Aspn	2.75E-11	1.838872221	0.269	0.125
Selenop	9.43E-10	2.013724176	0.322	0.203
Sfrp2	1.28E-09	1.969790787	0.267	0.141
Aqp1	1.79E-08	1.823691222	0.244	0.129
Ctss	2.16E-08	1.932997392	0.309	0.205
Col6a2	6.99E-08	1.742417922	0.244	0.136
Eln	1.37E-07	1.804318834	0.208	0.103
Rcn3	1.62E-07	1.779952233	0.238	0.134
Serpinf1	8.36E-07	1.900494745	0.28	0.194

Ptgis	3.53E-06	1.285682315	0.156	0.069
Itm2a	6.90E-06	1.646368949	0.204	0.118
Mfap5	8.54E-06	1.050285572	0.112	0.038
Cdh11	1.00E-05	1.516514972	0.2	0.116
Cthrc1	3.99E-05	1.653190345	0.225	0.152
Prrx1	9.72E-05	1.386330445	0.156	0.085
Trem2	0.000188988	1.342707629	0.171	0.103
Col8a2	0.000406145	1.082688835	0.109	0.051
Mxra8	0.001520039	1.650722069	0.213	0.165
Mfap2	0.00733422	1.154766775	0.124	0.08
Serpinh1	0.007871338	1.604420568	0.503	0.788
